# Convergent Evolution of Two Dopamine Receptor Genes: Repeated Evolution of Exon 6 Skipping in *Drd2*, and Repeated Deletion of Exon 6 in *Drd3*

**DOI:** 10.1007/s00239-025-10255-7

**Published:** 2025-06-09

**Authors:** Michael T. Peglar, Karl J. Fryxell

**Affiliations:** 1 School of Systems Biology, George Mason University, Manassas, VA USA; 2 Department of Biology; Math, Science, Business, and Engineering Division, Northern Virginia Community College, 8333 Little River Turnpike, Annandale, VA USA; 3 School of Systems Biology, and the Interdisciplinary Program in Neuroscience, George Mason University, Manassas, VA USA

**Keywords:** Alternative splicing, Exon skipping, Convergent evolution, Dopamine receptors

## Abstract

*Drd2* dopamine receptor mRNAs are alternatively spliced in rodents and primates by skipping exon 6 to produce the D2_S_ protein, or including exon 6 to produce the D2_L_ protein. These protein isoforms have differing roles in pre- vs. post-synaptic signaling, cytoplasmic vesicle processing, and calcium-mediated desensitization. Genetic alteration in the D2_S_/D2_L_ ratio affects human behavior and cognition at multiple levels, including working memory. Here we show that exon 6 originated early in vertebrate evolution, after the duplication and divergence of D2 and D4 dopamine receptor genes, but before the duplication and divergence of D2 and D3 dopamine receptor genes. Exon 6 encodes a relatively conserved sequence in the third cytoplasmic loop of the D2–D3 receptor. Its amino acid sequence is relatively short (24–33 amino acids), and is not strictly necessary for dopamine signal transduction. Exon skipping of *Drd2* exon 6 was not detectable in the brains of cyclostomes, sharks, fish, relatively primitive amphibians (*Xenopus, Notophthalmus*), relatively primitive reptiles (turtles), relatively primitive birds (ostrich), or relatively primitive mammals (monotremes and marsupials). However, exon skipping of *Drd2* exon 6 did occur at significant levels in the brains of more derived amphibians, reptiles, birds and mammals. Thus, skipping of *Drd2* exon 6 arose convergently and specifically in the more derived tetrapod lineages, none of which deleted this exon. In contrast, exon 6 was convergently deleted during *Drd3* evolution in an apparently random subset of the species of sharks, fish, amphibians, reptiles, birds, and mammals.

**Supplementary Information:**

The online version contains supplementary material available at 10.1007/s00239-025-10255-7.

## Introduction

Dopamine receptors belong to the rhodopsin branch of the G protein-coupled receptor (GPCR) superfamily (Fredriksson and Schiöth 2006). Dopamine receptors are divided into two main subdivisions. In mammals, the D1 subdivision includes dopamine receptors D1 and D5, which activate adenylate cyclase and are encoded by the *Drd1* and *Drd5* genes, respectively. The D2 subdivision includes dopamine receptors D2, D3 and D4, which act through multiple second messenger pathways, inhibit adenylate cyclase, and are encoded by the *Drd2*, *Drd3* and *Drd4* genes (Neve 2009). In the central nervous system (CNS) of vertebrates, dopamine receptors function primarily as neuromodulators that influence the excitability of neural pathways at both the presynaptic and postsynaptic levels (Neve 2009). In particular, the D2 dopamine receptor plays key roles in the regulation of signaling pathways that have been conserved from lamprey (a jawless fish) to humans. In the lamprey, these midbrain projections to the forebrain are involved in fine motor control, action selection and other functions (Robertson et al. 2012).

Mammalian *Drd1* and *Drd5* genes lack introns, but *Drd2*, *Drd3*, and *Drd4* genes have introns. Some of the intron locations are shared between subsets of these genes, while other intron locations are unique (Fryxell 1994). The intron locations have been conserved within the *Drd2* coding sequence of vertebrates (which encodes the D2 dopamine receptor protein), although some vertebrates may have an additional intron in the 5’ untranslated region. Thus, the correspondence of vertebrate *Drd2* exons between species is unambiguous, even though the numbering of these exons may differ in some publications or species. We will refer to any exon that is homologous to human *DRD2* exon 6 as "exon 6", and the conceptually-translated amino acid sequence as "the exon 6 amino acid sequence".

In humans, the exon 6 amino acid sequence encodes 29 amino acids in the third cytoplasmic loop of the D2 dopamine receptor. This includes binding sites for fatty acid binding protein (FABP3), which is involved in dendritic spine formation, and Rabex-5, which is involved in D2 receptor endocytosis (Shioda 2017; Shioda et al. 2017). The exon 6 amino acid sequence also contains a conserved arginine cluster that acts as an endoplasmic reticulum (ER) retention signal and modulates intracellular trafficking of the D2 dopamine receptor from the ER to the Golgi apparatus (Kubale et al. 2016). The D2_S_ protein isoform (which lacks the exon 6 sequence) and the D2_L_ protein isoform (which includes the exon 6 sequence) differ in their rates of calcium-mediated desensitization (Gantz et al. 2015), which is likely related to the differing rates of D2_S_ and D2_L_ endocytosis after dopamine binding, together with differences in cytoplasmic sorting and recycling (Sedaghat et al. 2006).

The *Drd2* exon 6 amino acid sequence is highly conserved in vertebrate species, with the proviso that this sequence is as long as 33 amino acids in length in some fish and amphibian species (Kubale et al. 2016). In some vertebrate species, *Drd2* exon 6 can be spliced out by exon skipping, thus eliminating the function(s) of the exon 6 amino acid sequence from a subset of the D2 receptor proteins. All species in which alternative RNA splicing of *Drd2* has been previously observed (primates, rodents, and the bullfrog) produce a long *Drd2* mRNA isoform that includes exon 6 (the *Drd2*_*L*_ mRNA, which encodes D2_L_ receptor proteins), and a short mRNA isoform that skips exon 6 (the *Drd2*_*S*_ mRNA, which encodes D2_S_ receptor proteins) (Dal Toso et al. 1989; Khan et al. 1998; Lindgren et al. 2003). Interestingly, previous studies also found that *Lithobates* (*Rana) catesbeiana* (bullfrog) does produce the *Drd2*_*S*_ mRNA isoform (Nakano et al. 2010), but another frog, *Xenopus laevis* (the African clawed frog) does not (Martens et al. 1993).

The D2_S_ isoform of the dopamine receptor functions in rodents and primates as a presynaptic dopamine autoreceptor, as shown by experiments with isoform-specific *Drd2* knockout mice, together with the D2 agonist quinpirole (Usiello et al. 2000; Lindgren et al. 2003). These studies showed that the D2_S_ receptor isoform was predominantly responsible for the regulation of tyrosine hydroxylase (TH) phosphorylation (which is presynaptic by definition, because TH catalyzes the rate-limiting step in dopamine biosynthesis). In contrast, the D2_L_ isoform was predominantly responsible for postsynaptic physiological responses, such as the phosphorylation of DARPP-32 (dopamine and cyclic AMP regulated phosphoprotein 32 kDa). Additional experiments confirmed that the presynaptic autoreceptor functions of D2_S_ help to regulate dopamine synthesis and release from presynaptic terminals (Usiello et al. 2000; Lindgren et al. 2003). These conclusions were tested by using mice who had been genetically engineered to specifically lack the D2_S_ isoform (D2_S_ -/- mice), and compared with mice who had been genetically engineered to specifically lack the D2_L_ isoform (D2_L_ -/- mice) (Radl et al. 2018). Low doses of quinpirole normally reduce motor activity in wild-type mice, due to an autoreceptor-mediated reduction in dopamine release. This reduced motor activity was absent in D2_S_ -/- mice but not in D2_L_ -/- mice (Radl et al. 2018). Moreover, quinpirole (in either low or high doses) failed to decrease the phosphorylation of tyrosine hydroxylase in D2_S_ -/- mice, but not in wild-type mice, nor in D2_L_ -/- mice (Radl et al. 2018). Taken together, these observations supported a major role for D2_S_ in presynaptic functions, including the regulation of dopamine synthesis (Radl et al. 2018). Moreover, immunocytochemical studies in rhesus monkeys confirmed that D2_S_-specific antibodies labeled dopaminergic cell bodies and axons, while D2_L_-specific antibodies labeled the postsynaptic targets of those axons in the striatum and nucleus accumbens (Khan et al. 1998).

In a related study, Centonze et al. found that the D2_S_ isoform was primarily responsible for the ability of quinpirole to inhibit glutamate-mediated spontaneous excitatory postsynaptic currents (Centonze et al. 2004). This reflects the fact that many dopaminergic neurons release both dopamine and glutamate, and also have presynaptic D2 autoreceptors that inhibit both dopamine release and glutamate release by that presynaptic terminal (Adrover et al. 2014). This ability of D2_S_ to modulate glutamate release is likely to be one of the most physiologically significant differences between the D2_S_ and D2_L_ isoforms.

The cognitive and behavioral significance of the D2_S_ and D2_L_ isoforms has been analyzed by studying human single nucleotide polymorphisms (SNPs) that specifically alter the splicing of *Drd2* transcripts. Two human SNPs, rs1076560 and rs2283265, are located in the introns before and after *Drd2* exon 6, respectively. Zhang et al. found that the minor alleles of these two SNPs reduced exon 6 skipping by more than twofold when both were present (Zhang et al. 2007). Interestingly, these minor alleles are in complete linkage disequilibrium with each other, meaning that the less common allele ("T") of rs1076560 is always found on the same chromosome as the less common allele ("T") of rs2283265 (Zhang et al. 2007). The functions of these two SNPs can be directly and separately tested in cell culture, by genetically engineering single DNA base substitutions in DRD2 minigenes expressed in HEK-293 cells (Zhang et al. 2007). The results showed that each of these minor alleles significantly reduced exon 6 skipping, without affecting the overall levels of expression of *Drd2* transcripts (Zhang et al. 2007). These results were also confirmed in an independent cell culture study in another laboratory (Cohen et al. 2015), which further showed that the "T" allele of rs1076560 prevented the binding of the putative mRNA splicing factor ZRANB2 (Loughlin et al. 2009). The minor alleles of these SNPs (rs1076560 and rs2283265) were also associated with highly significant reductions in the D2_S_/D2_L_ ratios in post-mortem samples from prefrontal cortex and striatum (Zhang et al. 2007), which has been confirmed in additional studies (Bertolino et al. 2009; Moyer et al. 2010; Blasi et al. 2015; Cohen et al. 2015), including a meta-analysis of five independent studies (Tunbridge et al. 2019).

At the level of human physiology and cognition, the minor alleles of these intronic SNPs were associated with greater neuronal activation during a working memory task in healthy adults (as measured by fMRI) (Zhang et al. 2007; Bertolino et al. 2009). These SNP alleles were also associated with reduced performance in working memory tasks (Zhang et al. 2007; Bertolino et al. 2009; Blasi et al. 2015), and reduced performance in attentional control tasks (Zhang et al. 2007). The minor alleles of rs2283265 and rs1076560 were also associated with relative impairments in making negative vs. positive decisions [i.e., avoidance vs. attraction (Frank and Hutchison 2009)]. This was expected because reduced feedback from presynaptic D2_S_ autoreceptors would elevate extracellular dopamine levels, whereas dips in extracellular dopamine levels are needed to identify and learn to avoid negative outcomes (Frank et al. 2007).

The minor allele of rs1076560 was also found to confer significantly increased risk of opioid dependence in two independent case–control studies (Clarke et al. 2014), and a significantly increased likelihood of alcoholism in a third study (Sasabe et al. 2007), and a significantly increased likelihood of cocaine abuse in a fourth study (Moyer et al. 2010), and a risk of cocaine abuse that was trending towards significance in a fifth study (Clarke et al. 2014). These results were consistent with results from animal models – for example, comparison of D2_L_ -/- vs. wild-type mice indicated that D2_L_ and D2_S_ dopamine receptors have distinct functional roles in drug addiction (cocaine, opiates) and in avoidance learning (Smith et al. 2002; Radl et al. 2018).

The cognitive and behavioral importance of the *Drd2*_*L*_/*Drd2*_*S*_ mRNA isoforms, together with the association of *Drd2*_*S*_ with dopamine autoreceptor functions, raises intriguing evolutionary questions. On the one hand, we know that the evolution of nervous systems has been accompanied by the elaboration of increasingly complex, and increasingly diversified, synaptic membrane-associated signaling complexes (Ryan and Grant 2009). The presynaptic microenvironment imposes unique constraints on the interaction of GPCR with calcium signaling (Dolphin and Lee 2020), and this may be relevant to the evolution of specialized D2 dopamine receptor isoforms, one of which is presynaptically localized in mammals. On the other hand, it remains unclear when (or why) the D2_S_ receptor isoform originated.

Here we show that the D2_S_ receptor isoform originated repeatedly, by convergent evolution of alternative RNA splicing. More specifically, exon skipping of Drd2 exon 6 evolved independently in each major group of tetrapods (mammals, reptiles, birds, and amphibians). This conclusion was based on quantitative analysis of brain RNA-Seq (RNA sequencing) data from all major subdivisions of the vertebrate lineages, together with a highly specific method of RNA splice junction analysis. In addition, we show by a similar analysis that exon 6 skipping in the *Drd3* gene (a paralog of *Drd2*) has remained barely detectable or non-existent in all vertebrates. But in the case of *Drd3*, a different sort of convergent evolution did occur, by genetic deletion of exon 6 via recombination between the two flanking introns.

## Results

### *Drd2 *Exon 6 Originated in Chordates, After the Duplication and Divergence of the *Drd2* and *Drd4* Dopamine Receptor Genes

**Fig. 1:**
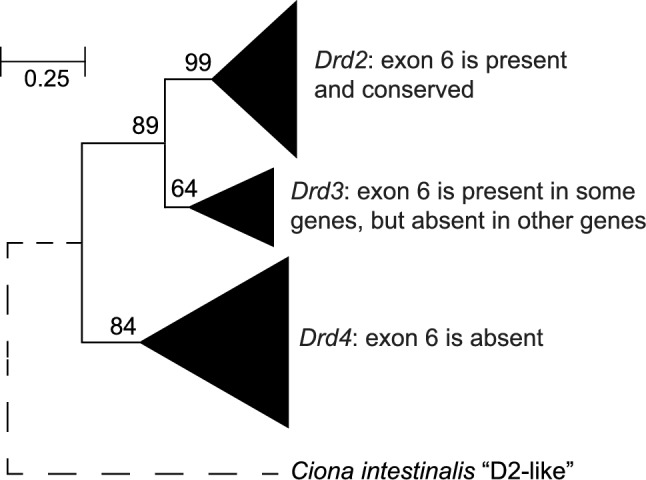
The major branches of the D2, D3, and D4 dopamine receptor gene family in lamprey, shark and fish genomes, illustrating the presence or absence of exon 6 in each branch. The sizes of the terminal triangles shown are proportional to the numbers of paralogs in each branch. This tree was based on a total of 57 genes from 10 species. The tree was generated by neighbor-joining and bootstrapping (see “Methods”). Bootstrap support (in percent) is shown next to each branch. Branch lengths are drawn to scale, in the units of the decimal fraction of amino acid substitutions per site (see scale bar), except that the root of the tree (shown as a dashed line) is not to scale. The root of the tree was generated by connecting the conceptually-translated product of the *Ciona intestinalis* "D2-like" gene (accession number XP_018667208.2) as an outgroup. A full expansion of the terminal triangles in this tree is shown in Supplementary Fig. S1

Any understanding of the evolution of alternative splicing of exon 6 must begin with an understanding of the origin and conservation of exon 6 in the context of the evolutionary history of dopamine receptors. We began by constructing protein trees of the D2-like dopamine receptors, based on the completed genomes of fish and chordate species. Our initial goal was to clarify when (and in which branches of this gene family) the exon 6 sequence originated. Figure 1 presents a condensed gene tree of all dopamine receptor genes related to D2, D3, and D4 dopamine receptors in chordate and fish species. The species names, gene sequences and gene symbols are listed in Supplementary Tables S2–S4, and the details of the tree branches are shown in Supplementary Fig. S1. Bootstrap analysis of this dataset showed strong bootstrap support (Fig. 1) for the conventional subdivisions of D2, D3, and D4 receptors (Fryxell 1994), as well as several additional conclusions (see below).

We found that the genome of the tunicate *Ciona intestinalis* (a primitive chordate) possessed only one gene that was annotated as belonging to the D2 subdivision of dopamine receptors. That annotation is questionable, because bootstrapped protein trees showed that the *C. intestinalis* "D2-like" protein sequence was an outgroup compared to vertebrate dopamine receptors, alpha-adrenergic receptors, and serotonin receptors (Supplementary Fig. S2). On the other hand, the homology of the *C. intestinalis* "D2-like" gene to *DRD2* was supported by the presence of an intron whose location was strictly conserved in alignments with human *DRD2.* Note, in this context the criteria for conservation (homology) of introns are that two intron locations align precisely within a multiple protein alignment, and are also spliced in a conserved phase of the open reading frame (Fryxell 1994). Note also that the retention of this one conserved intron location implies that neither the lineage leading to present-day *C. intestinalis* "D2-like", nor the lineage leading to human *DRD2*, have undergone any retrotransposition events in this gene since their last common ancestor (because retrotransposition events remove all ancestral introns). Because the other intron locations in *C. intestinalis* "D2-like" were not conserved with human *DRD2*, those appear to represent introns that were inserted during later stages of chordate/vertebrate evolution, as previously predicted (Fryxell 1994). Finally, the one conserved intron is the third intron in human *DRD2*, which lies within the coding sequence corresponding to the second cytoplasmic loop of both proteins. This conserved intron location does not correspond to either of the introns surrounding exon 6 in human *DRD2*. Rather, the introns surrounding exon 6 (and the protein coding sequence of exon 6 itself) were apparently inserted into the *Drd2* gene after the common ancestor with *C. intestinalis* (see below).

We found that the genome of *P. marinus* (sea lamprey) contained orthologs of the *Drd2* and *Drd4* genes, but no ortholog of the *Drd3* gene (see Supplementary Fig. S1). In contrast, the genomes of *Callorhinchus milii* (elephant shark), *Latimeria chalumnae* (coelacanth) and all actinopterygian fish species examined, contained orthologs of the *Drd2*, *Drd3*, and *Drd4* genes (Supplementary Fig. S1). Therefore, the duplication and divergence of *Drd2* and *Drd4* occurred within or prior to the Agnatha, while the duplication and divergence of *Drd2* and *Drd3* occurred somewhat later, around the time of origin of jawed fish (the Placoderms, who were ancestral to both Chondrichthyes and Osteichthyes).

Additional gene duplications resulted in additional *Drd2* paralogs that were unique to bony fish. In this regard, we found it helpful to distinguish between two major branches of fish *Drd2* paralogs, which we have labeled "D2α" and "D2β". These labels were simply used for convenience and to avoid confusion with the RNA splicing isoforms (see Supplementary Discussion and Supplementary Fig. S1). Briefly, the D2α branch was shared in common between all tetrapod D2 dopamine receptors and fish species. The D2β branch contained the remaining bony fish paralogs (see Supplementary Discussion and Supplementary Fig. S1). The members of the D2β branch were syntenic to each other, but not to the genes in the D2α branch [(Yamamoto et al. 2015); Peglar and Fryxell, unpublished results]. Overall, we found that exon 6 was present in the D2α, D2β, and D3 branches, but was absent from *C. intestinalis* "D2-like" and absent from all vertebrate genes encoding D4 receptors (Fig. 1). This implies that *Drd2* exon 6 originated after the common ancestor with *Ciona intestinalis*, but before the D2/D3 duplication, and therefore that exon 6 was originally shared in common between the newly-duplicated *Drd2* and *Drd3* genes.

### *Drd3 *Exon 6 was Deleted Several Times During Vertebrate Evolution, and the Entire *Drd3* Gene was Deleted in some Species

In principle, the specific deletion of a single intron is unlikely, because the genetic deletion of an entire intron together with the conservation of the protein coding sequences on both sides of the intron would require an extremely long deletion to occur with precisely-targeted breakpoints at both ends. Otherwise, the result would be a frameshift and/or premature stop codon, which likely explains why the intron locations in many genes have remained unchanged over vast stretches of evolutionary history. For example, five out of six of the introns in the central region of the triose phosphate isomerase gene have remained in the exact same location (including the same phase of the translation reading frame) since before the divergence of plants and animals more than one billion years ago (Marchionni and Gilbert 1986). Even the sixth intron was not an exception to this rule—rather, the sixth intron also remained in the same phase of the translation reading frame, but simply appeared to "slide over" by three codons due to an insertion or deletion within the protein coding sequence (Marchionni and Gilbert 1986).

**Fig. 2:**
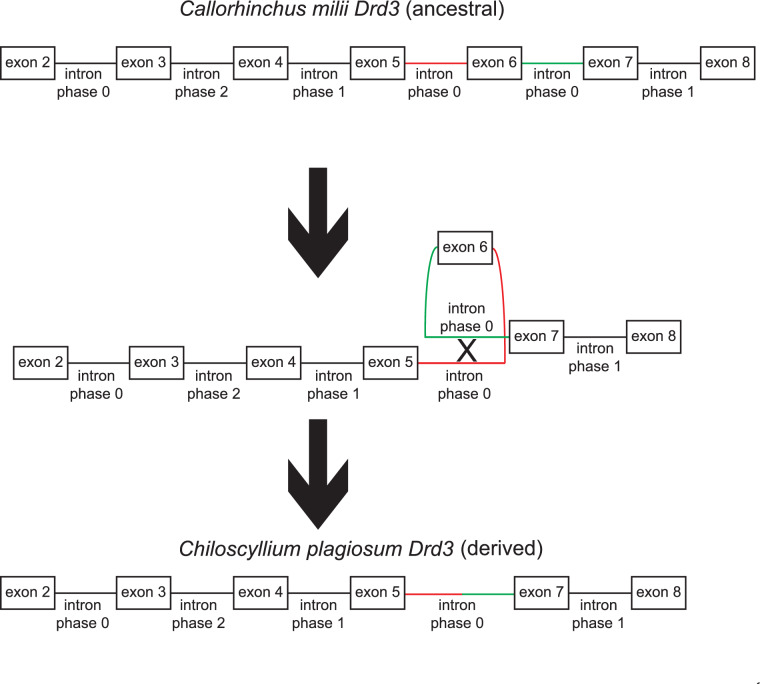
The exon/intron structure of the *Drd3* gene in the elephant shark (*Callorhinchus milii*) and the white-spotted bamboo shark (*Chiloscyllium plagiosum*). The ancestral exon/intron structure in *Callorhinchus milii Drd3* was modified in *Chiloscyllium plagiosum Drd3*, evidently by recombination between the pair of phase 0 introns, thus simultaneously deleting one intron and exon 6. Exon numbers were assigned by homology to mouse (*Mus musculus*) *Drd3* (see “Methods” section entitled “Identification of conserved introns”). Intron phase numbers refer to the intron locations, in terms of the phase of the translational reading frame in which mRNA splicing occurs (phase 0 = splicing between codons) (Color figure online).

Given these considerations, we were surprised to find that, in the exon/intron structure of the *Drd3* gene of *Latimeria chalumnae* (a sarcopterygian fish), the phase 0 intron downstream of exon 6 was missing. Further investigation confirmed that the phase 0 intron that is normally present upstream of exon 6 was still present in its expected location (with respect to the protein coding sequence). However, the amino acid sequence encoded by exon 6 itself was absent, as was the phase 0 intron downstream of exon 6. The remainder of the *Drd3* protein coding sequence (corresponding to the third cytoplasmic loop, followed by transmembrane segments 6 and 7) was present and intact. These facts strongly suggested that a single genetic deletion had been caused by recombination between the two flanking phase 0 introns (see Fig. 2). These sorts of deletions can occur, for example, by genetic recombination between two copies of transposable elements (Sen et al. 2006).

**Fig. 3:**
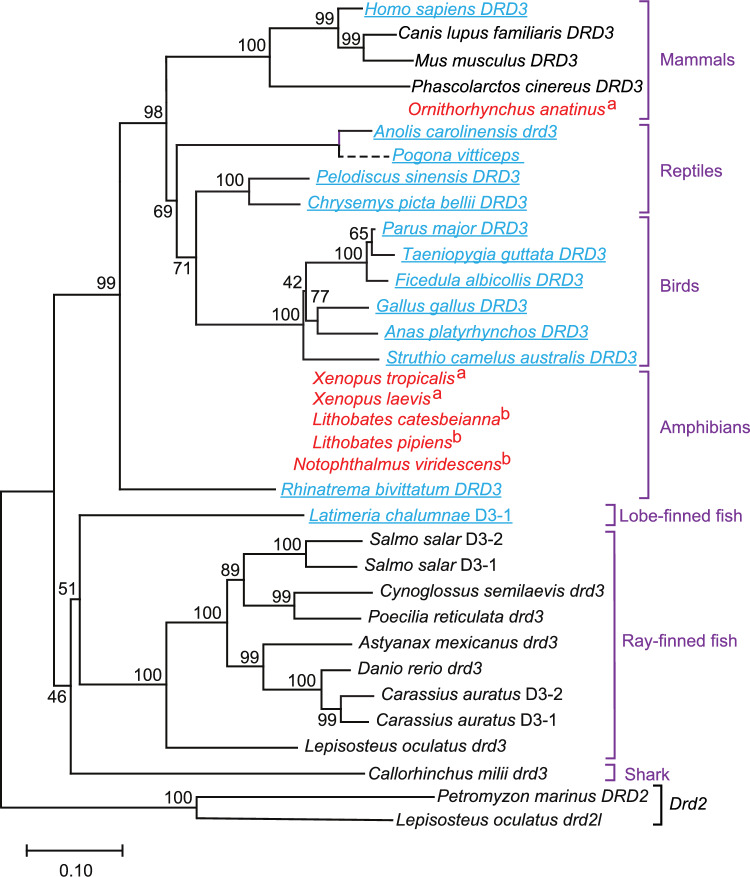
Gene family tree for vertebrate D3 dopamine receptor genes. The status of the *Drd3* gene, and its exon 6 (in each species), as well as a *Drd3* pseudogene, are represented in this figure as follows: Genes/species in standard font represent *Drd3* genes in which exon 6 was present and flanked by a pair of phase 0 introns. Genes/species in underlined fonts (blue and underlined in the online version) represent *Drd3* genes in which exon 6 was deleted, evidently by recombination, leaving behind only one phase 0 intron (see Fig. 2). The dashed branch leading to *P. vitticeps* represents a *Drd3* pseudogene (in which exon 6 had also been deleted, leaving only one phase 0 intron behind). Our conclusion that this was a pseudogene was based on the presence of an in-frame stop codon within the third cytoplasmic loop, which we found in the genomic sequence and independently confirmed in RNA-Seq data, together with the deletion of ~ half of the protein coding sequence (shown in the Ref-Seq genome, and which we confirmed by genomic BLAST experiments) and the transcription of the remainder at very low levels (see Supplementary Table S5). Species with no gene name (red species names in the online version) and no connecting branches represent genomes in which the entire *Drd3* gene was absent. The conclusion that the *Drd3* gene was absent was supported by evidence indicated by superscripts as follows: (a) species that lacked the *Drd3* gene in their genome sequence and also lacked detectable *Drd3* mRNA in RNA-Seq libraries from brain tissue; (b) species with no currently available genome sequence, but RNA-Seq brain libraries in which *Drd3* sequences were not detectable with full-length query sequences from closely-related species (see “Methods” for further details). This tree was generated by neighbor-joining and bootstrapping (see “Methods”). Bootstrap support (in percent) is shown next to each branch. Branch lengths are generally drawn to scale, in the units of the decimal fraction of amino acid substitutions per site (see scale bar). However, the branch length for the *P. vitticeps* pseudogene could not be drawn to scale, because some of the expected upstream exons were not present in the genome sequence. Accordingly, the *P. vitticeps* pseudogene was also excluded from our bootstrap calculations. The root of this tree was generated by connecting the conceptually-translated products of the *Petromyzon marinus DRD2* gene (XP_032820438.1) and the *Lepisosteus oculatus drd2l* gene (XP_015224221.1) as out-groups (as shown). In cases where no gene symbol was available from the RefSeq annotations, we used informal gene abbreviations, as follows (gene abbreviation followed by the corresponding protein sequence accession numbers): *C. auratus D3-1*, XP_026057526.1; *C. auratus D3-2*, XP_026093276.1; *L. chalumnae D3-1*, XP_014350627.1; *S. salar D3-1*, XP_045558612.1; *S. salar D3-*2, XP_014037004.2. Further details are in Supplementary Table S3 (Color figure online)

We also discovered additional species whose *Drd3* gene had one phase 0 intron downstream of exon 5 (rather than a pair of phase 0 introns), similar to *Latimeria chalumnae*. This pattern was found in the amphibian *Rhinatrema bivittatum*, and in *Homo sapiens*, and in all of the birds and reptiles in our dataset (shown in blue in Fig. 3). In each of these cases, protein alignments confirmed that no amino acid sequences homologous to exon 6 remained in the *Drd3* genes, indicating that exon 6 had been deleted by genetic recombination between the two flanking phase 0 introns (see below). The other mammals in our dataset retained both of the phase 0 introns and also retained the *Drd3* exon 6 coding sequence (shown in black in Fig. 3). This configuration was also found in the *Drd2* and *Drd3* genes of actinopterygian fish and in at least one shark species (shown in black in Fig. 3). However, we subsequently discovered that exon 6 in *Drd3* had been deleted in several other shark species (see below).

Even larger *Drd3* deletions were evident in several amphibian genomes, in which we found that *Drd3* dopamine receptor sequences were entirely absent by DNA and RNA analysis (see Fig. 3 and the legend to Fig. 3). Other authors have shown by molecular evidence that the surviving clades of amphibians share a single monophyletic origin (Venkatesh et al. 2001). Therefore, the presence of a *Drd3* gene in at least one surviving amphibian species (*Rhinatrema bivittatum*, Fig. 3) indicates that the *Drd3* gene was present in the common ancestor of all amphibians, but was subsequently deleted from numerous amphibian species. Likewise, we found no *Drd3* dopamine receptor sequences by DNA or RNA analysis in a primitive mammalian species (the platypus, *Ornithorhynchus anatinus*; see Fig. 3). In this case, the presence of *Drd3* in several other mammalian species indicates that *Drd3* was present in the common ancestor of all mammals, but was specifically lost in *Ornithorhynchus anatinus*. Finally, we also found that the *Drd3* gene has mutated into a pseudogene in the reptile *Pogona vitticeps*, but not in the other reptiles in our dataset (see Fig. 3 legend and Supplementary Table S5). Taken together, these results indicate that *Drd3* gene function was lost entirely in multiple independent events during vertebrate evolution—and that even in some of the species in which the *Drd3* gene was retained, the exon 6 portion of *Drd3* has been lost.

### The Exon 6 Amino Acid Sequence was Well Conserved in D2 Dopamine Receptors, but the Exon 6 Amino Acid Sequence in D3 Dopamine Receptors Diverged More Rapidly

The *Drd2* dopamine receptor genes of shark and fish contain a short 6th exon (28–33 codons) that is bounded on both sides by phase 0 introns (i.e., mRNA splicing occurs between codons in both introns). In most cases, the *Drd3* dopamine receptor genes of sharks and fish also contained a short (24–28 codons) exon 6 that was bounded on both sides by phase 0 introns. However, *Drd4* genes did not have any sequence that was homologous to *Drd2* exon 6, as shown by the absence of both homologous intron locations in all *Drd4* genes, and by the absence of any amino acid sequences that were detectably similar to the *Drd2* exon 6 amino acid sequence (see below and Table 1).

**Table 1:** Phylogenetic distribution of protein sequences similar to *DRD2* exon 6 of *Canis lupis familiaris*

	*Drd2*	*Drd3*	*Drd4*	Other genes
Chondrichthyes	16	0	0	0
Sarcopterygii	4	0	0	0
Actinopterygii	401	0	0	0
Amphibians	26	0	0	0
Reptiles	56	0	0	0
Birds	409	0	0	0
Mammals	266	0	0	0
Invertebrates	0	0	0	0
Fungi	0	0	0	0
Protists	0	0	0	0
Bacteria	0	0	0	0
Totals	1,178	0	0	0

In species for which multiple protein isoforms from the same gene were present in the results, we counted each gene only once in these subtotals. The identities of poorly annotated genes were clarified by reciprocal BLAST searches (see Methods)

**Fig. 4:**
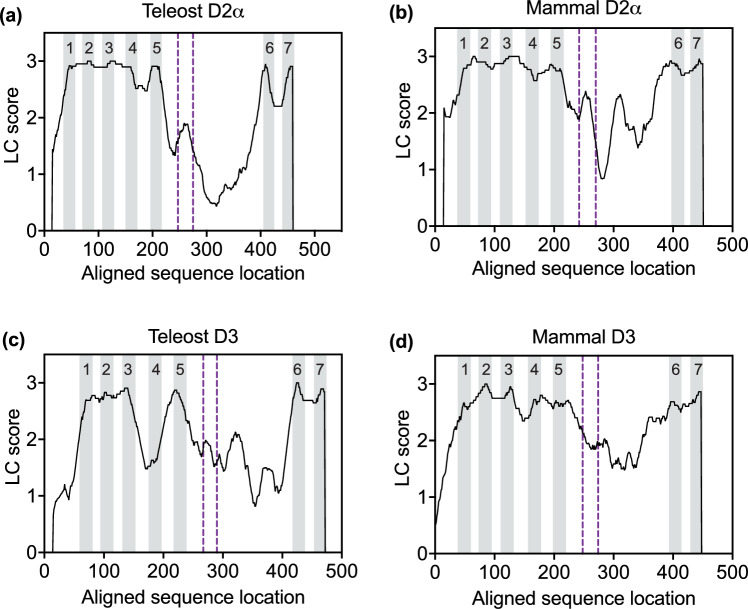
Sliding window analysis of local protein sequence conservation in *Drd2* and *Drd3* genes of fish and mammals. The locations of the seven protein transmembrane segments are numbered and shaded in grey. The boundaries of exon 6 are shown by vertical dashed lines (purple in the online version). The algorithm used for sliding window analysis of local protein sequence conservation was described in Methods. All of the *Drd2* sequences shown in this figure belong to the D2α branch of the gene family (see Fig. 7 and Supplementary Fig. S1). **A** Teleost D2α. Sequences used in this panel were from the following species: *Astyanax mexicanus* (2 genes), *Carassius auratus* (2 genes)*, Cynoglossus semilaevis*, *Danio rerio* (2 genes), *Poecilia reticulata,* and *Salmo salar*. **B** Mammalian D2α. Sequences used in this panel were from the following species: *Camelus ferus*, *Canis lupus familiaris*, *Homo sapiens*, *Microcebus murinus* (XP_012608768.1), *Monodelphis domestica* (XP_016289342.1), *Mus musculus*, *Myotis davidii* (XP_006177161.1), *Ornithorhynchus anatinus*, *Phascolarctos cinereus,* and *Sus scrofa* (NP_001231182.1). **C** Teleost D3. Species were the same as in panel (**A**). Most teleost species in this panel had only one *Drd3* gene, except *Carassius auratus*, which had two *Drd3* genes. **D** Mammalian D3. Mammalian species were the same as in panel (**B**), except that *O. anatinus* was omitted because it does not have a *Drd3* gene, and *H. sapiens* was omitted because it does not have *Drd3* exon 6 (see Fig. 3). For further details, see Supplementary Tables S2 and S3 (Color figure online)

Based on a sliding-window analysis of amino acid sequence conservation (Fig. 4A), we found that the D2 dopamine receptors of teleost fish contained a distinct peak of amino acid sequence conservation that was located near the center of *Drd2* exon 6. Note that the location of exon 6 in Fig. 4 is indicated by vertical dashed lines (purple in the online version), and the locations of protein transmembrane segments (which are conventionally numbered from left to right) is indicated by vertical gray bars. Thus the "third cytoplasmic loop" corresponds to the large white space between transmembrane segments 5 and 6. The computational algorithm used to draw these graphs quantified sequence conservation in 30-amino acid windows and plotted the scores of each window with respect to the sequence address of the center of that window (see “Methods”). Thus a peak of sequence conservation near the center of exon 6 indicates that the conservation of the exon 6 amino acid sequence was greater than the conservation of adjacent sequences in the third cytoplasmic loop. On the other hand, the sequence conservation scores in the 3rd cytoplasmic loop tended to be somewhat lower than in the transmembrane segments, due to the generally higher frequency of amino acid substitutions, insertions and deletions in the 3rd cytoplasmic loop.

This evolutionary pattern (i.e., the conservation of the *Drd2* exon 6 amino acid sequence, relative to adjacent sequences) was analyzed separately in mammals. The results confirmed that mammals also show a peak of sequence conservation near the center of the *Drd2* exon 6 (Fig. 4B). Moreover, teleost *Drd3* genes also showed a distinct peak of amino acid sequence conservation within exon 6 (Fig. 4C). On the other hand, when preparing to analyze mammalian *Drd3* genes, we first had to omit the human *Homo sapiens* (because the human *Drd3* gene lacks exon 6; see previous section), and also omit the platypus *Ornithorhynchus anatinus* (which does not have a *Drd3* gene, see Fig. 3). With these caveats, we found that mammalian *Drd3* did not show a clear peak of sequence conservation in exon 6 (Fig. 4D). However, the quantitative conservation scores within mammalian *Drd3* exon 6 were similar to those in teleost *Drd3* (Fig. 4C). Taken together, these results provide evidence that the exon 6 amino acid sequence has been maintained by purifying selection in *Drd2* and *Drd3,* although the exon 6 sequence differs between these genes (see below).

The genomic and phylogenetic distribution of the *Drd2* exon 6 amino acid sequence was explored with a BLASTP (protein BLAST) search of the non-redundant version of Genbank, using the exon 6 amino acid sequence (29 amino acids) of a mammalian *Drd2* gene (*Canis lupus familiaris*) as the query sequence (Table 1). An E-value of 0.05 was used as the significance threshold after correction for multiple comparisons. The lowest-scoring significant match was identical to the query sequence at 12 locations (41% identity). Overall, the *Drd2* exon 6 amino acid sequence was found to be present (conserved) in essentially all vertebrate D2 dopamine receptors (Table 1), with the proviso that lamprey (*P. marinus*) D2 scored just below the threshold of significance (not shown). No significant (or nearly significant) matches were found in the "D2-like" gene of *Ciona intestinalis* (a primitive chordate), nor in D3 dopamine receptors of any species, nor in any other genes (see Table 1, and “Methods”).

An additional BLASTP search with a more primitive query sequence (*P. marinus Drd2* exon 6) gave D2-specific matches to other lampreys, sharks, bony fish, amphibians, birds, reptiles and marsupial mammals. In this case, the *Drd2* exon 6 sequences of placental mammals scored just below the threshold of significance (not shown).

Given that the lamprey genome represents the state of the *Drd2* gene prior to the evolutionary origin of the *Drd3* gene, we next asked what happened to the exon 6 amino acid sequence during the first stages of evolution after the duplication and divergence of *Drd2*/*Drd3* genes? This question was addressed by analysis of exon 6 in the *Drd2* and *Drd3* genes of *Callorhinchus milii* (a relatively primitive shark) and *Lepisosteus oculatus* (a relatively primitive bony fish). We found that the *Drd2* exon 6 amino acid sequences of these two species were still 100% identical to each other (29/29 amino acid identities). In contrast, the *Drd3* exon 6 amino acid sequences of those same two species had already become highly diverged (6/28 = 21% amino acid identities).

We followed up this observation by using the *Drd3* exon 6 amino acid sequence of *Callorhinchus milii* as a query sequence for BLASTP. However, that query sequence yielded no significant matches (other than itself), in all of Genbank! This raised the question why *C. milii* did not match other shark *Drd3* genes? When we examined recently annotated shark *Drd3* genes (*Hemiscyllium ocellatum*, *Chiloscyllium plagiosum*, *Rhincodon typus*, and *Carcharodon carcharias*) and matched their *Drd3* exons with orthologous exons in *C. milii*, we found that none of the four additional shark species had a putative exon 6 in their *Drd3* genes, nor did they have any other exons that were 20–30 codons in length and bounded by phase 0 introns. The most detailed empirical evidence of the exact *Drd3* exon/intron structure was present in *C. plagiosum,* which had both a genomic scaffold and 100% RNA-Seq coverage of the *Drd3* transcript. That data proved that *C. plagiosum* had only one phase 0 intron downstream of *Drd3* exon 5, as expected if exon 6 had been deleted by recombination between two ancestral phase 0 introns (Fig. 2). In *C. plagiosum Drd3, a*ll the other introns, exons, and splicing locations (with respect to the translational reading frame) matched the ancestral configuration seen in *C. milii Drd3* (Fig. 2).

Taken together, these results showed that the *Drd3* exon 6 amino acid sequence diverged from *Drd2* rather quickly after the duplication and divergence of *Drd2*/*Drd3*; and that the exon 6 of *Drd3* was sometimes deleted by recombination between the two flanking introns. In contrast, the *Drd2* exon 6 amino acid sequence was immediately and strongly conserved after the duplication and divergence of *Drd2*/*Drd3*, and *Drd2* exon 6 has apparently never been deleted.

The subsequent evolution of *Drd3* exon 6 in vertebrates was characterized by using the amino acid sequence of the zebrafish (*Danio rerio*) *Drd3* exon 6 as the query sequence (24 amino acids). The lowest-scoring significant match was identical to the query sequence at 14 locations (58% identity). We obtained significant matches to the *Drd3* genes of bony fish, but not to the *Drd3* genes of tetrapods, lampreys or sharks, nor to the *Drd2* genes of any species. Conversely, when we used the amino acid sequence of a mammal (*Canis lupus familiaris*) *Drd3* exon 6 as the query sequence (26 amino acids), we obtained significant matches to the *Drd3* genes of most mammalian species (with the exception of mammalian species that lack exon 6, as noted above), but not to the *Drd3* genes of any fish, amphibian, bird, or reptile; nor to the *Drd2* genes of any species. In this case, the lowest-scoring significant match was identical to the query sequence at 17 locations (65% identity).

Taken together, these results confirmed that *Drd3* genes do contain exon 6 in some vertebrate species. Where present, *Drd3* exon 6 was characterized by sequence conservation within taxonomic classes such as bony fish and mammals. However, the amino acid sequence encoded by *Drd3* exon 6 diverged significantly between taxonomic classes, while the amino acid sequence encoded by *Drd2* exon 6 did not.

### Measurements of the Levels of *Drd2*_*L*_ and *Drd2*_*S*_ mRNA Isoforms in the CNS of Vertebrate Species

**Fig. 5:**
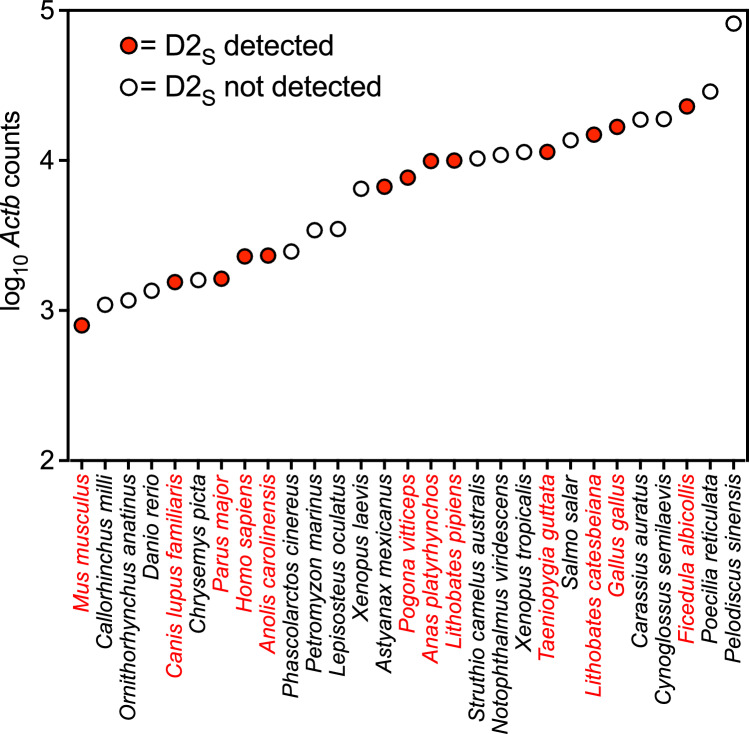
Presence or absence of the *Drd2*_*S*_ isoform, in relation to the sequencing depth of RNA-Seq libraries in various species. The Y-axis of this graph represents the total sequencing depth, expressed as the total number of *Actb* (β-actin) sequencing reads (that matched our *Actb* ej-SRA BLAST query sequences), in all of the RNA-Seq libraries that we used from that species. The X-axis of this graph represents the species analyzed, sorted in order from species with the lowest number of *Actb* sequencing reads on the left, to species with the highest number of *Actb* sequencing reads on the right. Filled circles (solid red circles and red species binomens in the online version) represent species in which both *Drd2*_*L*_ and *Drd2*_*S*_ isoforms were present. Open circles represent species in which only the *Drd2*_*L*_ isoform was present. Overall, there was no relation between the sequencing depth (number of *Actb* sequencing reads) and the apparent presence or absence of the *Drd2*_*S*_ isoform. RNA-Seq libraries and accession numbers are listed in Supplementary Table S1 (Color figure online)

We used *Drd2* genomic and cDNA sequences to identify *Drd2* exons 5, 6 and 7 for each species in this study (listed along the X-axis of Fig. 5). The same procedure was followed for exons 3 and 4 of the *Actb* genes of these species, which were used as controls to determine the relative mRNA levels and RNA-Seq library quality. With these exon sequences, we designed search sequences [also known as BLAST queries] that covered selected exon junctions, with 15 nucleotides on each side of the exon boundary (see “Methods”). We refer to these BLAST query sequences as "Exon Junction RNA Sequences" (ej-RNAs), because they were used for BLAST searches of digitally-archived RNA-Seq libraries. This allowed us to identify RNA-Seq reads that included a particular splice junction, and thus to quantify the relative abundance of *Drd2*_*L*_ and *Drd2*_*S*_ mRNA isoforms in the brain tissues of 29 species. We counted only RNA-Seq reads that matched the ej-RNA query sequence with a perfect 30/30 match. This choice was supported by control experiments that established the specificity and sensitivity of this procedure (see below).

**Fig. 6:**
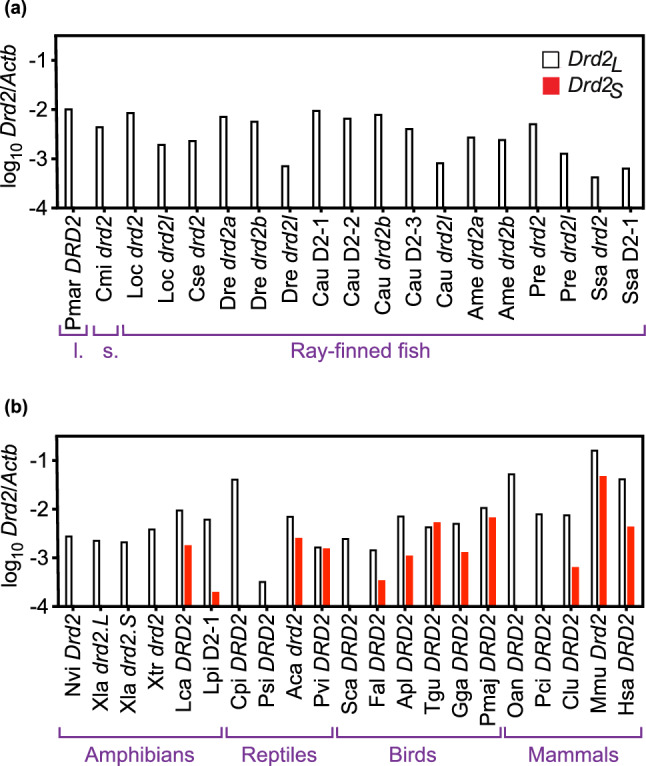
Expression of *Drd2* dopamine receptor isoforms in vertebrate brain tissue. Expression values were quantified by counting RNA-Seq reads with perfect matches to ej-RNAs (see “Methods”). White bars represent *Drd2*_*L*_ isoforms, and red bars represent *Drd2*_*S*_ isoforms. These *Drd2* expression values were normalized by dividing by the corresponding RNA-Seq read counts for β-actin (*Actb*), and expressing the ratio as the log_10_ of the *Drd2*/*Actb* ratio. **A** fish. **B** tetrapods*.* Species in this figure are grouped by taxonomic classes, and the classes were labeled by their common names (l. = lamprey, s. = shark). Species labels consist of a 3–4 letter abbreviation of the species binomen, followed by the gene symbol or informal gene abbreviation (“D2” followed by an integer). Abbreviations of species binomens are as follows (listed in alphabetical order): Aca, *Anolis carolinensis*; Ame, *Astyanax mexicanus*; Apl, *Anas platyrhynchos*; Cau, *Carassius auratus*; Clu, *Canis lupus familiaris*; Cmi, *Callorhinchus milli*; Cpi, *Chrysemys picta*; Cse, *Cynoglossus semilaevis*; Dre, *Danio rerio*; Fal, *Ficedula albicollis*; Gga, *Gallus gallus*; Hsa, *Homo sapiens*; Lca, *Lithobates catesbeiana*; Loc, *Lepisosteus oculatus;* Lpi, *Lithobates pipiens*; Mmu, *Mus musculus*; Nvi, *Notophthalmus viridescens*; Oan, *Ornithorhynchus anatinus*; Pci, *Phascolarctos cinereus*; Pmaj, *Parus major*; Pmar*, Petromyzon marinus*; Pre, *Poecilia reticulata*; Psi, *Pelodiscus sinensis*; Pvi, *Pogona vitticeps*; Sca, *Struthio camelus*; Ssa, *Salmo salar*; Tgu, *Taeniopygia guttata*; Xla, *Xenopus laevis*; Xtr, *Xenopus tropicalis*. The *Astyanax mexicanus drd2l* gene was omitted from this figure, because its expression levels were too low to generate RNA-Seq matches to isoform-specific ej-RNAs (we were able to confirm that this gene is expressed, by using other query sequences, see Supplementary Methods and Supplementary Table S5). The informal gene abbreviations used in this figure are as follows (abbreviation followed by gene ID or Genbank accession number): Cau D2-1, 113,058,429; Cau D2-2, 113,115,093; Cau D2-3, 113,081,886; Lpi D2-1, sequence assembly from this study (Supplementary Table S2); Ssa D2-1, 106,582,624. Additional details are listed in Supplementary Tables S1, S2 and S6–S8 (Color figure online)

Beyond *Drd2*_*L*_ and *Drd2*_*S*_, we also found additional *Drd2* splice variants in platypus (*Ornithorhynchus anatinus*) and in bird species. In general, these additional splice variants resulted in length variation of exons 5 and/or 7. For purposes of the present report, these isoforms were included in the subtotals for *Drd2*_*L*_ or *Drd2*_*S*_, based on the presence or absence of exon 6. This required the design and use of additional ej-RNAs for these species (see Supplementary Table S6). We also quantified cytoplasmic β-Actin (*Actb*) mRNA with ej-RNAs, both as an estimate of the effective sequencing depth (relative amount of useful RNA sequence information) within each RNA-Seq library (Fig. 5), and to normalize (determine the relative levels of) the *Drd2* isoform counts between species (Fig. 6).

### The Impact of Sequencing Depth on the Detection of the *Drd2* Isoforms

Because we used ej-RNAs to quantify splicing by matching RNA-Seq reads across a diverse array of genomes, this raised the issue of whether the sequencing depth of these diverse 133 brain RNA-Seq libraries (from 29 species) had been sufficient to allow the reliable detection of both *Drd2* mRNA isoforms. In theory, species in which the available RNA-Seq libraries contained relatively few total sequence reads, and/or relatively short read lengths, could have failed to match one of our ej-RNA query sequences simply by chance. In practice, we found that the *Drd2*_*L*_ isoform was detectable in all tested species, and the apparent presence or absence of the *Drd2*_*S*_ mRNA isoform was unrelated to the sequencing depth of that library (assessed by the number of matches to an *Actb* ej-RNA) (Fig. 5). More specifically, species with the lowest sequencing depth (those on the left in Fig. 5) did not show a bias towards having fewer species with *Drd2*_*S*_ [species with *Drd2*_*S*_ are shown as filled symbols in Fig. 5 (red in the online version)]. Similarly in terms of the relative levels of *Drd2* transcripts, those species with lower normalized *Drd2*_*L*_ transcript levels (represented by shorter white bars in Fig. 6) did not show a bias towards having fewer species in which *Drd2*_*S*_ was detectable (*Drd2*_*S*_ isoforms are represented by red bars in Fig. 6). Both lines of evidence indicate that our ability to detect *Drd2*_*S*_ isoforms was not limited by the available RNA sequencing depth in the species tested.

A related issue was that a few of the genes in this study had relatively low numbers of RNA-Seq read counts (< 10 *Drd2*_*L*_ RNA-Seq reads for a few of the genes; see Supplementary Table S8), which limited the precision of our estimates of the expression levels of these particular genes (Fig. 6). Although quantitative analysis of gene expression levels was not a primary goal of this paper, nevertheless any genes with relatively low numbers of *Drd2*_*L*_ RNA-Seq reads could still raise questions about our ability to detect the corresponding *Drd2*_*S*_ isoforms. We note that 3 of these genes were found in fish species (*Danio rerio*, *Salmo salar, Lepisosteus oculatus*) in which RNA-Seq reads were detected for multiple *Drd2* paralogs, and the total number of *Drd2* RNA-Seq reads was substantially greater than 10. None of those paralogs had any *Drd2*_*S*_ counts, supporting the general absence of exon 6 skipping in fish. The only other species with relatively low *Drd2*_*L*_ counts (*Callorhinchus milli*) also had no *Drd2*_*S*_ RNA-Seq reads, and that conclusion was consistent with results from another cartilaginous fish (the lamprey, *Petromyzon marinus*) which also lacked *Drd2*_*S*_ RNA-Seq reads.

Another potential limitation on our detection of *Drd2* isoforms was the potential presence of sequencing errors, annotation errors, genetic polymorphisms, and so on, which may have reduced our ability to find matches to our ej-RNAs. We undertook a detailed analysis of these issues, which showed that these issues were detectable, but were not frequent enough to affect our conclusions (see Supplementary Methods).

### Confirmation of the Genetic Identity of RNA-Seq Reads Retrieved via ej-RNAs

Because we used ej-RNAs to quantify splicing across a diverse array of species, it was important to confirm whether the RNA-Seq reads matching these ej-RNAs were reliably derived from the correct genes in all of these species. Accordingly, we retrieved all of the 2,198 complete RNA-Seq reads that matched the ej-RNAs used to quantify the *Drd2*_*L*_ and *Drd2*_*S*_ isoforms in the 29 species that we analyzed (Supplementary Table S8). Many of these species had several *Drd2* paralogs and/or mRNA isoforms, as a result of which a total of 94 ej-RNAs were tested (Supplementary Table S6), of which 57 ej-RNAs matched RNA-Seq reads with a perfect (30/30) match to the corresponding ej-RNA. The total read length of the retrieved RNA-Seq reads was 236,984 nucleotides, which is equivalent to an average read length (per RNA-Seq read) of 108 bases. Additional sequence information from each RNA-Seq read (beyond the match to the ej-RNA) was used to determine whether the retrieved RNA-Seq reads actually represented legitimate transcripts from the *Drd2* gene of the species in question (see below).

Of the 2,198 retrieved RNA-Seq reads, 2,134 (97%) were simply *Drd2* transcripts. Some of these differed from the expected sequence by ≤ 5 scattered base substitutions that may have represented RNA sequencing errors (often in the first 10 bases), RNA polymerase errors, or allelic polymorphisms. The remaining 65 RNA-Seq reads (3% of the total) contained sufficient perfectly matched sequence adjacent to the ej-RNA (48–276 bases) to confirm their origin from the appropriate *Drd2* isoform, but also contained additional sequences that fell into several categories. The first category consisted of rearranged *Drd2* sequences from the same species—but from the wrong DNA strand, and/or separated from the matched portion by a lengthy gap, and/or derived from a different portion of the *Drd2* transcript (6 RNA-Seq reads). These rearranged *Drd2* sequences presumably represented PCR artifacts such as strand switching. The second category were chimeric reads that contained other gene sequences from the same species (4 RNA-Seq reads, all from *C. auratus*). The third category were chimeric reads that contained other gene sequences from other species (plants, bacteria, virus, etc.: 17 RNA-Seq reads). The fourth category were vector and/or barcode sequences that represented failures of data processing to remove these expected sequences from the dataset (14 RNA-Seq reads). Finally, the fifth category contained additional sequences that were too short to be identified by BLAST (24 RNA-Seq reads).

As itemized above, none of these categories of chimeric reads represented a significant fraction of our dataset. All contained sufficient sequence information to identify them as having been derived from transcripts of the designated isoform of *Drd2*. We concluded that all of the RNA-Seq reads that matched our ej-RNAs were correctly identified, and did include the desired splice junction from the correct gene, and could therefore be counted in our analysis. The only caveat was that ~ 3% of our RNA-Seq reads were chimeric, due to incomplete data processing and issues during library construction such as PCR artifacts.

### The *Drd2*_*L*_ isoform was Present in all Tested Species, but the *Drd2*_*S*_ Isoform was Present Only in a Subset of the Tetrapods

Our results showed that the 29 species we tested fell into two categories: species that expressed substantial levels of both *Drd2*_*L*_ and *Drd2*_*S*_ isoforms (12 species), and species that expressed only the *Drd2*_*L*_ isoform (17 species) (Figs. 5, 6).conclusion was supported by additional experiments to assess the adequacy of the sequencing depth, as well as the genetic identity of the retrieved RNA-Seq reads (see above).

BLAST read counts for the *Drd2*_*L*_ and *Drd2*_*S*_ isoforms were normalized to read counts for *Actb* and presented in a log-transformed bar graph (Fig. 6). The results show that the *Drd2*_*L*_ isoform was present in the brains of all tested species, but the *Drd2*_*S*_ isoform was not present in the brains of lamprey (*P. marinus*), shark (*C. milii*), or any bony fish species (Fig. 6A). Among tetrapods, some species in each taxonomic class expressed both *Drd2*_*L*_ and *Drd2*_*S*_ isoforms, while other species in the same class expressed only the *Drd2*_*L*_ isoform (Fig. 6B)*.* For amphibians, both ranid frog species [*Lithobates (Rana) catesbeiana* and *Lithobates (Rana) pipiens*] expressed both *Drd2*_*L*_ and *Drd2*_*S*_. However, the tropical frog species tested (*Xenopus tropicalis* and *Xenopus laevis*) and the spotted newt (*Notophthalmus viridescens*) expressed only the *Drd2*_*L*_ isoform. For reptiles, both hard-shell turtle (*Chrysemys picta*) and soft-shell turtle (*Pelodiscus sinensis*) lacked the *Drd2*_*S*_ isoform, while the squamate lizards (*Anolis carolinensis* and *Pogona vitticeps*) did have the *Drd2*_*S*_ isoform. Among the birds, ostrich (*Struthio camelus*) expressed only *Drd2*_*L*_, but all other bird species tested expressed both *Drd2*_*L*_ and *Drd2*_*S*_ isoforms (Fig. 6B)*.* Among the mammals, platypus (*Ornithorhynchus anatinus*) and koala bear (*Phascolarctos cinereus*) expressed only the *Drd2*_*L*_ isoform, but dog (*Canis lupus*), mouse (*Mus musculus*) and human (*Homo sapiens*) expressed both *Drd2*_*L*_ and *Drd2*_*S*_ isoforms (Fig. 6B)*.*

**Fig. 7:**
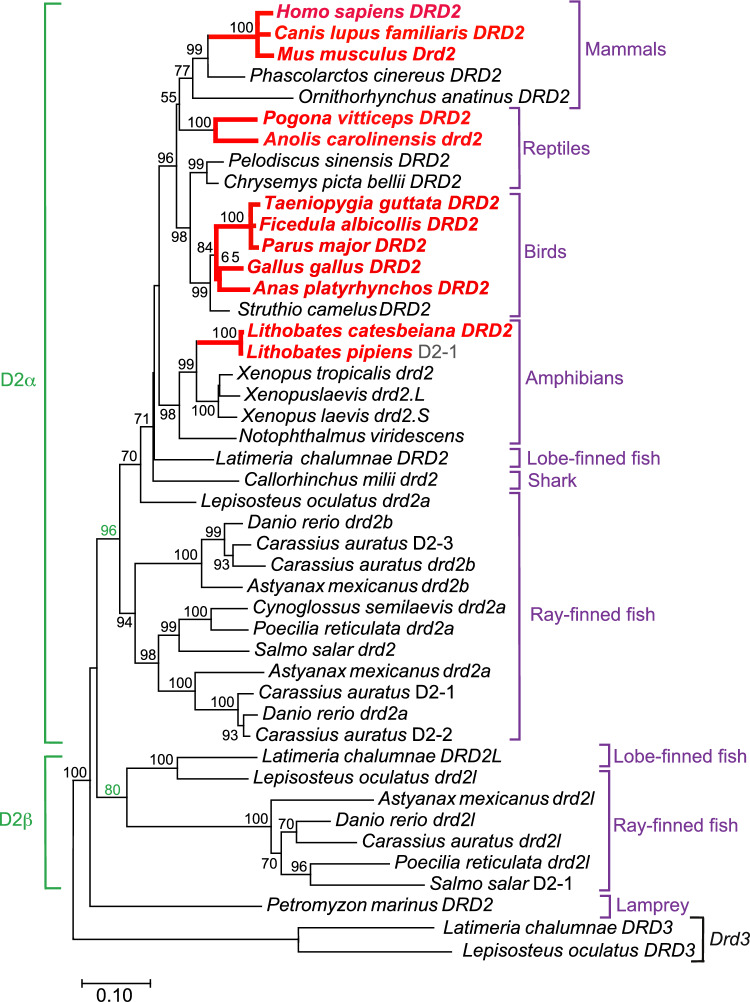
Protein family tree for vertebrate D2 dopamine receptors. Bold font and connecting branches (red in the online version) represents genes/species in which *Drd2* exon 6 skipping was present. In all other genes/species, *Drd2* exon 6 skipping was absent. Note that *Drd2* intron locations were conserved on both sides of exon 6. Brackets on the left of this figure (green in the online version, including green bootstrap support values) indicate the D2α and D2β subdivisions of D2 receptors. Bootstrap values for each internal branch are shown, except that bootstrap values < 50% were omitted. Brackets and labels on the right of this figure (purple in the online version) indicate the common names of taxonomic classes. Protein trees were generated by neighbor-joining and bootstrapping (see “Methods”). Bootstrap support (in percent) is indicated next to each branch. Branch lengths are drawn to scale, in the units of the decimal fraction of amino acid substitutions per site (see scale bar). The root of the tree was generated by connecting the outgroups *L. chalumnae D3-1* (accession number XP_014350627.1) and *L. oculatus drd3* (accession number XP_015196681.1) as shown. Definitions of the informal gene abbreviations used in this figure are as follows (abbreviation followed by the protein accession number): *C. auratus* D2-1, XP_026082110.1; *C. auratus* D2-2, XP_026138158.1; *C. auratus* D2-3, XP_026109573.1; *L. pipiens* D2-1, sequence assembly from this study (Supplementary Table S2); *S. salar* D2-1, XP_014021327.1. Additional details are listed in Supplementary Table S2 (Color figure online)

We have illustrated these results in a bootstrapped protein tree of D2 dopamine receptors (Fig. 7). In this figure, we used bold nodes (red in the online version) and bold branches (red in the online version) to label those species and lineages that did exhibit exon 6 skipping (i.e., both *Drd2*_*L*_ and *Drd2*_*S*_ isoforms were present). The species and lineages without exon 6 skipping were not attributable to any disruption of *Drd2* exon/intron structure, because we found that *Drd2* exon/intron structure was strictly conserved (see above). Nor was the absence of *Drd2* exon 6 skipping due to the variations (between species) in the sequencing depth of the RNA-Seq libraries (Figs. 5, 6).

Overall, the phylogenetic pattern shown in Fig. 7 suggests that skipping of *Drd2* exon 6 evolved once in each of the major tetrapod groups (amphibians, reptiles, birds, and mammals), and was conserved in each of those groups after it evolved. Moreover, the skipping of *Drd2* exon 6 was confined to tetrapods, and evolved only in the D2α branch of the gene family (Fig. 7).

### Measurements of the Levels of *Drd3*_*L*_ and *Drd3*_*S*_ mRNA Isoforms in the CNS of Vertebrate Species

We also analyzed our dataset for evidence of *Drd3* exon 6 skipping. This analysis was limited by the fact that many of the species in our dataset were missing the *Drd3* gene, or were missing exon 6 of the *Drd3* gene (see above). We did find occasional traces of *Drd3* exon 6 skipping in a few fish species, but in levels so low that we doubt whether they were significant. More specifically, we found one single RNA-Seq read that skipped exon 6 (per species) in *L. oculatus*, *D. rerio*, and *A. mexicanus*. Other shark (*C. milli*) and fish (*C. auratus*, *P. reticulata*, *S. salar*, *C. semilaevis*) species showed no evidence of *Drd3* exon 6 skipping. Amphibians, reptiles, and birds could not be tested for *Drd3* exon 6 skipping, either because they lacked *Drd3* exon 6, or because they lacked the entire *Drd3* gene (Fig. 3). None of the mammals that we tested showed any evidence of *Drd3* exon 6 skipping.

## Discussion

### The Evolutionary History of a Protein Module

The starting point of our analysis was the "D2-like" gene of *Ciona intestinalis*, a chordate that is closely related to vertebrates (Delsuc et al. 2006; Putnam et al. 2008; Satou et al. 2019)*.* The *C. intestinalis* "D2-like" gene was clearly homologous to vertebrate *Drd2*, although *C. intestinalis* "D2-like" may be more accurately characterized as an early stage in the evolution of catecholamine receptors (see “Results”). In any case, we found that the *Drd2* exon 6 amino acid sequence was not present in the *C. intestinalis* "D2-like" gene. Moreover, the pair of phase 0 introns that demarcate exon 6 were also not present in the *C. intestinalis* "D2-like" gene.

The next step in the evolution of *Drd2* and related genes was represented by lampreys such as *Petromyzon marinus*. *P. marinus* is a jawless fish belonging to the cyclostomes, also known as agnathan vertebrates (Smith et al. 2013). The *P. marinus* genome contained orthologs of the *Drd2* and *Drd4* genes, but not of the *Drd3* gene (Supplementary Fig. S1). *P. marinus Drd2* did have a recognizable exon 6 amino acid sequence, as well as the pair of phase 0 introns that demarcate exon 6, but *P. marinus Drd4* lacked both of these introns and the exon 6 sequence. This apparently abrupt origin of a novel amino acid sequence, bordered by a pair of new phase 0 introns, suggests the possibility that exon 6 might have originated by recombination (exon shuffling) with other genes. We cannot rule out this possibility, but our sequence comparisons did not yield any statistically significant evidence of evolutionary precursors from other genes (Table 1).

### Repeated Deletion of Exon 6 from *Drd3 *Genes, but not From *Drd2* Genes

The next step in the evolution of *Drd2* and related genes was represented by sharks such as *Callorhinchus milii* [a relatively primitive shark; (Venkatesh et al. 2014)]. We found that *C. milii* had orthologs of *Drd2*, *Drd3*, and *Drd4*. The new gene here was *Drd3*, which clearly represented a duplicated copy of *Drd2* (including exon 6 and the pair of phase 0 introns). However, the sequence of *C. milii Drd3* exon 6 had diverged considerably from *Drd2* exon 6. Moreover, examination of four additional shark species revealed that exon 6 had been deleted from their *Drd3* genes in all four species, with a single phase 0 intron remaining behind in each case. An example is shown in Fig. 2. We also found additional examples of *Drd3* exon 6 deletion in some (but not all) species of bony fish, amphibians, reptiles, birds, and mammals [species in which *Drd3* exon 6 was deleted are underlined in Fig. 3 (and blue/underlined in the online version)]. The absence of exon 6 from *Drd3* in the lobe-finned fish *L. chalumnae* was particularly interesting (Fig. 3), because *L. chalumnae* is closely related to the tetrapods. But the surviving species of lobe-finned fish are sister taxa of the tetrapods (rather than being direct ancestors of the tetrapods), and the tetrapods are monophyletic (Venkatesh et al. 2001), from which we can infer that the first tetrapods must have retained the ancestral (fish) *Drd3* exon/intron structure. This also means that the genetic deletion of *Drd3* exon 6 occurred repeatedly—in some (but not all) sharks, and in some lobe-finned fish, and in some amphibians, and in (some, possibly all) birds/reptiles, and in some (but not all) mammals.

Why was exon 6 deleted so frequently from *Drd3* genes (Fig. 3) but not from *Drd2* genes (Table 1; Fig. 1)? Where *Drd3* exon 6 was present, we found that it was invariably rather short (24–28 codons) and surrounded on both sides by phase 0 introns. Where exon 6 was absent, we found that there was invariably one phase 0 intron remaining behind (i.e., a phase 0 intron that now was directly adjacent to exons 5 and 7). These facts implicated recombination between the two phase 0 introns as the molecular mechanism of exon 6 deletion in *Drd3* (Fig. 2). In theory, such crossovers may have been facilitated by the pairing of repetitive DNAs that happened to be shared between those two introns (Sen et al. 2006), although different repetitive DNAs may have been involved in each case. Overall, the existence of these repeated instances of deletion of exon 6 from *Drd3* genes, along with equally numerous instances in which this exon was retained (Fig. 3), suggests that the functions of exon 6 were somewhat dispensable in the context of D3 dopamine receptors, but on the other hand there was no strong advantage of deleting *Drd3* exon 6. In any case, the deletion of exon 6 by recombination between the two flanking introns (Fig. 2) would not disrupt the open reading frame, nor would it prevent signal transduction by the dopamine receptor (Gantz et al. 2015).

In contrast, exon 6 was never deleted from *Drd2* genes (see Fig. 1; Table 1). We found that the amino acid sequence encoded by exon 6 was more strongly conserved in *Drd2* genes than in *Drd3* genes (see “Results”). Moreover, previous experiments with D2 isoform-specific knockout mice indicated that there was a major presynaptic role for the D2S isoform (but not the D2L isoform), while the D2L isoform was required for most of the motor and cellular responses to acute cocaine administration (Radl et al. 2018). Moreover, D2L-specific knockout mice displayed defects in learning to avoid aversive stimuli such as an electric shock (Smith et al. 2002). Such behaviors would certainly be of survival value. Taken together, these observations indicate that the mammalian D2S and D2L isoforms are functionally distinct from each other, and that neither isoform is dispensable. Thus, our observation that exon 6 was never deleted from *Drd2* (in any species in our study) probably reflects ongoing natural selection for an essential function(s) of exon 6 in the context of *Drd2* genes.

### *Drd2* Exon 6 Skipping was not Present in CNS of Fish, Shark, or Lamprey

Skipping of exon 6 evidently evolved long after the origin of exon 6, in fact it was observed only in a subset of tetrapod species. More specifically, the *Drd2*_*L*_ splice isoform was easily detectable in the brains of every species tested, but the *Drd2*_*S*_ isoform was not found in RNA-Seq libraries from the brains of lamprey, shark, actinopterygian fish or sarcopterygian fish (Fig. 6A). Previous authors have reported that *Drd2*_*S*_ mRNA was specifically detectable within the hypothalamic/pituitary axis of goldfish during their brief mating season (Popesku et al. 2011). We did not attempt to test this. However, at the present level of analysis (with whole brain tissue collected at unstated times of year), we found no evidence of *Drd2* exon 6 skipping in goldfish (*Carassius auratus*) brain tissue, which included tests of all five *Drd2* paralogs from that species.

### *Drd2* Exon 6 Skipping Evolved Convergently in at Least Four Tetrapod Lineages

As stated above, we found that the *Drd2*_*S*_ mRNA isoform was not detectable in the central nervous system (CNS) of any fish species that we examined (Fig. 6A). We infer that the absence of D2_S_ in the CNS was the ancestral condition in bony fish, and by extension was also the ancestral condition in the first tetrapods. This is the most parsimonious explanation for several reasons. First, given that D2_S_ was consistently absent from the CNS of fish and other primitive vertebrates (Fig. 7), parsimony would favor the short isoform also being absent from their descendants (the first tetrapods). Secondly, if one were to hypothesize that the presence of the short isoform had been ancestral among tetrapods, one would then have to also postulate that D2_S_ had been gained once (in the common ancestor of all tetrapods) and lost six or more times (among the various black branches in Fig. 7). It would be more parsimonious to postulate to the contrary, that the absence of D2_S_ was ancestral among tetrapods, because under that hypothesis, D2_S_ would have been gained only four times and never lost (within this tree; see Fig. 7). Third, if the absence of D2_S_ were postulated to have been ancestral among tetrapods, this would also be more consistent with results from comparative genomics, which indicate that exon skipping was generally less frequent among primitive tetrapods than among more advanced groups of mammals (Kim et al. 2007; Barbosa-Morais et al. 2012). Fourth, if exon skipping to form D2_S_ were actually ancestral, then one would expect this trait to have been retained in some primitive tetrapods—such as turtles (among the reptiles), or monotremes (among the mammals), but we found no such examples (see below).

More specifically, we found that *Drd2* exon 6 skipping was absent from *Xenopus*, who belong to the most primitive group of frogs (Archaeobatrachia). In contrast, *Drd2* exon 6 skipping was present in frogs such as *Lithobates* (*Rana)*, who belong to a recently-evolved group (the Neobatrachia) that underwent rapid diversification after the Cretaceous-Paleogene extinction event (Feng et al. 2017).

Among the living reptiles, turtles have traditionally been considered to be the most primitive group, although data from recent molecular and fossil morphological studies have established that turtles are, in fact, the sister clade of the Archaeosaurs (birds, crocodiles), from whom they began to diverge in the mid-Permian, while Squamates (lizards, snakes) form a separate clade that split off in the early to mid-Permian (Lyson et al. 2013; Schoch and Sues 2015; Li et al. 2018). This divergence order is consistent with our D2 protein tree, in which turtles did cluster with birds, while lizards formed a separate group (Fig. 7). On the other hand, the basic turtle body plan has remained relatively unchanged since the Triassic [see pp. 112–117 in (Romer 1966)], while Squamates (lizards and snakes) continued to diversify into a larger and more diverse species group. In this study, we found that turtles lacked *Drd2* exon 6 skipping, but lizards did have *Drd2* exon 6 skipping (Fig. 7).

Among the birds, paleognaths are the most primitive group, based on the reptile-like configuration of their bony palate (Houde 1988). Ostriches are the most basal birds within the paleognaths, based on genomic sequence comparisons (Cloutier et al. 2019). We found that ostriches did not skip *Drd2* exon 6, but more derived bird species did have *Drd2* exon 6 skipping (Fig. 7).

Among the mammals, monotremes (*Ornithorhynchus anatinus*) and marsupials (*Phascolarctos cinereus*) are more primitive than placental mammals, based on their reproductive physiology and their basal positions in the mammalian phylogenetic tree (Veyrunes et al. 2008). We found that monotremes and marsupials did not skip *Drd2* exon 6, but more derived mammals (eutherians) did have exon 6 skipping in their CNS (Fig. 7).

We conclude that exon-skipping of *Drd2* exon 6 to produce the D2_S_ protein isoform evolved independently and convergently in at least four different lineages (amphibians, reptiles, birds, and mammals). The clades that do have *Drd2* exon 6 skipping (the bold branches in Fig. 7 (red in the online version)) are well separated from each other in taxonomic terms (Fig. 7). Such repeated rounds of convergent evolution imply that exon-skipping of *Drd2* exon 6 may have been advantageous in tetrapods. Furthermore, the tetrapod lineages that did evolve exon-skipping of *Drd2* exon 6 seem to have been generally more successful than the tetrapod lineages that did not, by the usual criteria of "evolutionary success" (giving rise to a larger number of surviving descendant species and colonizing a wider variety of environments). Admittedly, this is a provocative possibility that requires further examination, as it seems questionable whether such a broad correlation with evolutionary success could be attributable to the splicing of one small exon.

### The Evolutionary Significance of D2 Exon 6 Skipping

In molecular terms, several considerations suggest that the ancestral *Drd2*/3 exon 6 was "preadapted" to exon skipping (or exon deletion). First, exon 6 was relatively short, and it was bounded by introns that were in the same phase of the translational reading frame (phase 0 introns). That meant that translation could continue uninterrupted, regardless of whether exon 6 was intact, or was skipped, or was deleted. Secondly, the amino acid sequence encoded by exon 6 represented protein binding sites that were involved in protein processing and recycling, but were not strictly necessary for signal transduction by dopamine receptors (Sedaghat et al. 2006). That meant that exon 6 skipping or deletion might not be deleterious. Third, exon skipping could be easily initiated, if base substitutions created a single binding site for an RNA splice suppressor (Wang and Burge 2008). Fourth, we have presented evidence in this report that the genetic deletion of *Drd3* exon 6 did occur via recombination between the flanking introns (Fig. 2), and this recombination occurred repeatedly in evolutionary history (Fig. 3). This was arguably another consequence of the "preadaptation" of exon 6 to exon deletion or exon skipping.

Within the set of species analyzed in this report, we found that exon 6 skipping did evolve repeatedly, and once it did evolve, it was apparently never lost (Fig. 7). But that does not necessarily mean that the skipping of *Drd2* exon 6 would have always been advantageous in all species. It is striking that the skipping of *Drd2* exon 6 *never* evolved in fish, sharks, or basal tetrapods, over a time period of hundreds of millions of years (Fig. 7). This suggests that the prior evolution of additional, interacting proteins may have been a prerequisite for producing selective advantages of the D2_S_ isoform. This same theme (the prior evolution of interacting proteins) was a major influence on the coevolution of gene families (Fryxell 1996).

In modern mammals, the primary function of the D2_S_ protein isoform is as a presynaptic dopamine autoreceptor (Khan et al. 1998; Usiello et al. 2000; Lindgren et al. 2003; Zhang and Sulzer 2012). Within the presynaptic terminal, the physiological functions of the D2_S_ autoreceptor are complex and differ somewhat from its postsynaptic functions. Presynaptic autoreceptor functions include decreasing dopamine exocytosis via modulation of Ca^++^ and K^+^ currents (Zhang and Sulzer 2012; Ford 2014), increasing dopamine reuptake by the dopamine transporter (DAT), decreasing local dopamine synthesis (Zhang and Sulzer 2012), and modulating the localization of D2_S_ receptors via protein kinase C signaling (Luderman et al. 2015). These and other presynaptic functions require the coordinated actions of many proteins (Pinheiro and Mulle 2008), far beyond the capabilities of one small exon in the *Drd2* gene. In this sense, the prior evolution of mechanisms of presynaptic regulation of neurotransmitter release may have been a prerequisite for effective selection in favor of a presynaptic dopamine receptor isoform. Likewise, during the evolution of neurotransmitters and second messenger pathways, evidence suggests that the prior evolution of related genetic functions was a prerequisite for the duplication and divergence of neurotransmitter receptor genes that took advantage of those evolutionary opportunities (Fryxell and Meyerowitz 1991; Fryxell 1996). In the present case, we propose that the absence of exon 6 (and its ER retention signal) would have allowed D2_S_ receptors to leave the ER and thus be more efficiently transported to axons and presynaptic terminals, while D2_L_ receptors continued to be retained within the rough ER, from which location they could reach membranes in the neuronal cell body and dendrites (Hudson AD, Murphy RL, Peglar MT, and Fryxell KJ, unpublished data). We note that little rough ER is present in axons (Öztürk et al. 2020).

Comparative studies have shown that exon skipping is the most common type of alternative RNA splicing in vertebrates, while other types of alternative splicing (such as intron retention) are more common in fungi, protists, and plants (Kim et al. 2007; McGuire et al. 2008). In terms of exon skipping in vertebrates, there are more genes (and more exons) involved in exon skipping in the brain than in any other tissue, and more in humans > other primates > other mammals > birds > reptiles > amphibians (Kim et al. 2007; Barbosa-Morais et al. 2012). Comparative analysis has further shown that vertebrate gene expression consisted of tissue-specific patterns of expression that were conserved across species. However, exon skipping showed the opposite pattern—namely species-specific patterns of exon skipping that were shared between tissues within a species (Barbosa-Morais et al. 2012), as if each species (or lineage) evolved new mechanisms of exon skipping that had been broadly deployed in multiple tissues of that species. Taken together, these considerations suggest that exon skipping has contributed significantly to the evolution of biological complexity in vertebrate animals (Ule and Blencowe 2019).

The evolution of a newly-derived type of exon skipping, or a newly-evolved mechanism of presynaptic regulation (or both) could increase the potential for the future evolution of other types of exon skipping and neural circuits. As Lynn Helena Caporale once remarked, "chance favors the prepared genome" (Caporale 1999). Put another way, the "evolution of evolvability" (Radman et al. 1999) may have influenced the adaptive radiations of tetrapod groups. This could result, for example, from: (i) the cumulative selective advantages of regulated exon-skipping in an increasing number of genes (Herbert and Rich 1999; Barbosa-Morais et al. 2012), and/or (ii) the deployment of presynaptic regulatory mechanisms across an increasing number of neural circuits (Pinheiro and Mulle 2008). Either (or both) of these could explain why the convergent evolution of *Drd2* exon 6 skipping occurred primarily in the more derived tetrapod lineages. The end result was that a small protein module that originated in jawless cartilaginous fish has now become regulated by alternative RNA splicing, and is deployed in a way that has acquired broad significance in mammalian physiology and cognition (Centonze et al. 2004; Zhang et al. 2007; Frank and Hutchison 2009; Radl et al. 2018).

## Methods

### Species and Tissues

We chose taxa for mRNA splicing analysis that had available RNA-Seq libraries (Wang et al. 2009) in the National Center for Biotechnology Information (NCBI), sequence read archive (SRA) (Leinonen et al. 2011). Where possible, we selected libraries prepared from whole brain tissue. For human (*Homo sapiens*) and chicken (*Gallus gallus*) we had to use libraries from specific brain areas (Supplementary Table S1). In the case of painted turtle (*Chrysemys picta*), the tissues of origin were not recorded in the Sequence Read Archive, but we chose 2 out of 12 of the RNA-Seq libraries from this species, which did have relatively high ratios of *Drd2*/actin and calcium–calmodulin-dependent protein kinase II (*CaMKII*)/actin, and were therefore likely to have been derived from brain tissue (Zalcman et al. 2018). For the soft-shell turtle (*Pelodiscus sinensis*), we used two libraries from whole embryos of various developmental stages (see Supplementary Table S1), in order to provide additional data on *Drd2* splicing in turtles.

The goldfish (*Carassius auratus*) is a domesticated species that was derived from the common carp (*Cyprinus carpio*) more than 1000 years ago. Both species are allopolyploid (Chen et al. 2019). The RefSeq genome sequence for *C. auratus* was based on the "Wakin" strain, which was sequenced at the National Human Genome Research Institute (Bethesda, MD, USA) (Chen et al. 2019). We compared this to the only available RNA-Seq data from adult brain tissue of the goldfish, which came from two Chinese studies—one from the South China Agricultural University (Guangzhou, China), and the other from the Chinese Academy of Sciences, Institute of Hydrobiology (Wuhan, China). Both Chinese studies utilized unstated goldfish strains. *C. auratus* is known to have considerable genetic heterogeneity between strains (Braasch 2020; Kon et al. 2020), so these strains may have differed significantly from the "Wakin" strain. In the course of analyzing the RNA-Seq data, we found a group of 126 RNA-Seq reads that defined an additional, novel *C. auratus Drd2* paralog—a paralog that was not present in the RefSeq "Wakin" genome sequence. We did not include this novel paralog in the present study, because the strain of origin was unknown, and because this paralog was not present in the reference genome (see Supplementary Methods).

In general, we attempted to obtain samples from as many vertebrate classes as possible, preferably with two or more species from each class. The final data set used for mRNA analysis included 133 RNA-Seq libraries from 29 species in 9 vertebrate classes (Supplementary Table S1). For 26 of these species, we were able to use complete (or nearly complete) genome sequences in the NCBI Reference Sequence Database (RefSeq) (McGarvey et al. 2015; O’Leary et al. 2016), along with curated annotations of genomic and mRNA sequences. These were used to locate open reading frames and exon/intron splice junctions in the analyzed genes (see below). For the remaining 3 species, genome sequences were not available, but we were able to infer the locations of open reading frames and exon/intron splice junctions based on additional information that is described below in the section entitled "mRNA isoform-specific ej-RNAs”.

### Genetic Nomenclature

Due to the variety of gene names and gene symbols used in the species examined here, this report utilized mammalian genetic nomenclature (mouse or human) for general comments about groups of dopamine receptor genes. Specific sequences (particularly in the figures and tables) were identified with the gene symbols accepted for that species (as defined in RefSeq), and shown in italics. If the RefSeq annotation did not include a formal gene symbol for a particular gene, then we used informal gene abbreviations (shown in roman font), which were defined in the figure legends and Supplementary Tables. The Supplementary Tables cross-referenced all gene symbols and informal gene abbreviations, as well as sequence accession numbers, gene IDs, published gene names, species common names and species scientific names. See Supplementary Tables S2–S4.

### Identification of Outlier Genes and Pseudogenes

Putative dopamine receptor genes were excluded from our analysis if the conceptually translated protein sequence did not have seven transmembrane segments, and/or did not group with other dopamine receptors by bootstrap analysis. Transmembrane segments were identified and counted with the computer program "TransMembrane segment prediction by Hidden Markov Models" (TMHMM v. 2.0, DTU Health Tech, Denmark Technical University, Lyngby, Denmark).

That said, it is also true that two of the genes we identified as outliers were included in selected portions of our analysis, for the following reasons: The first outlier that we used was *Pogona vitticeps Drd3*, which our sequence analysis showed was a pseudogene, due to the presence of premature stop codon(s) (see legend to Fig. 3). That result was informative when included in our *Drd3* gene tree (Fig. 3), because it supported the general conclusion that *Drd3* functions were frequently lost in vertebrates. The second outlier that we used was the *Ciona intestinalis* gene annotated as "D2-like", which we concluded represented an early stage in the evolution of catecholamine receptors, because that gene was equally distantly related to vertebrate dopaminergic, adrenergic, and serotonergic receptors (Supplementary Fig. S2), and shared only one conserved intron location with those genes (see “Discussion”). Those properties made *Ciona intestinalis* "D2-like" an appropriate sequence for locating the root of our dopamine receptor gene trees (see Fig. 1; Supplementary Fig. S1).

Another questionable gene was *Astyanax mexicanus drd2l*. This gene did contain a conserved open reading frame with seven transmembrane segments, and it did cluster with dopamine receptors by bootstrap analysis (Fig. 7). This gene was evidently expressed at very low levels, and we confirmed that it was expressed by searching RNA-Seq libraries with customized query sequences that were specific for exons 5, 6, or 7 (not shown). Unfortunately, these expression levels were too low to be detected by isoform-specific ej-RNAs. Therefore, this gene was excluded from our isoform analysis (see legend to Fig. 6).

Additional outlier genes were excluded entirely for reasons discussed in Supplementary Methods (see also Supplementary Fig. S2, Table S5).

### Construction of Protein Trees

Protein sequences were aligned by Clustal X in the MEGA software package (Kumar et al. 2018; Stecher et al. 2020). Multiple protein sequence alignments were used for the construction of gene trees by the neighbor-joining method (Saitou and Nei 1987), which was performed in MEGA with 10,000 bootstrap iterations (Felsenstein 1985). Evolutionary distances in phylogenetic trees were computed with the Jones Taylor Thornton (JTT) matrix-based method (Jones et al. 1992).

In general, our protein trees were based on the same 29 species we used for RNA-Seq analysis, with two exceptions. First, as mentioned above, the "D2-like" gene of the tunicate *Ciona intestinalis* was used as an outgroup to locate the root in gene trees of vertebrate D2, D3, and D4 dopamine receptor genes. This gene could not be used in our analysis of exon 6 splicing, because it did not contain a sequence similar to the amino acid sequence encoded by exon 6, nor did it contain the homologous intron locations that define exon 6 in other species. Secondly, we added coelacanth (*Latimeria chalumnae*) dopamine receptors to our protein trees, to help to clarify the evolutionary history of *Drd2* paralogs in lobe-finned vs. ray-finned fish. However, these *L. chalumnae* genes were not used in our analysis of exon 6 splicing, because there was no available RNA-Seq data (SRA libraries) from *L. chalumnae* brain tissue*.*

D3 protein trees (Fig. 3) presented another problem, because the genomes of some of our species did not appear to have *Drd3* genes. We therefore invested some effort in verifying whether or not this was really the case. For species with no annotated *Drd3* gene, or with no completed genome sequence, we analyzed their RNA-Seq libraries by SRA BLAST, using a complete *Drd3* mRNA sequence from the most closely related species available as the query sequence. In the case of amphibians that were missing *Drd3*, we used the *Drd3* mRNA sequence from the limbless amphibian *Rhinatrema bivittatum* (two-lined caecilian) as the query sequence.

### Identification of Conserved Introns

In order to ‘compare apples to apples’ in the analysis of RNA splicing, it was necessary to establish that we were comparing the splicing of homologous exons (i.e., exons bordered by conserved intron locations). The task of identifying homologous exons was complicated by the differing numbering conventions for those exons in various species. Also, introns can be gained or lost during evolutionary history, and DNA sequences within introns are often poorly conserved (Graur 2016). Therefore, we used the following criteria to establish the existence of a conserved intron location: (i) exact alignment of the relevant exon–intron boundaries across multiple protein sequence alignments, and (ii) strict conservation of the phase of RNA splicing with respect to the open reading frame (Fryxell 1994), and further that (iii) intron locations within the protein coding sequence correspond to structurally and functionally equivalent protein domains. In order to evaluate these criteria, multiple protein sequence alignments were performed in MEGA (see above), and exon–intron boundaries within each sequence were visualized with the RefSeq Gene Viewer (McGarvey et al. 2015; O’Leary et al. 2016), and/or the NCBI Spline Tool (Kapustin et al. 2008). These software packages provided simultaneous graphical views of exon/intron junctions and the corresponding cDNA and protein sequences (and thus the location and phase of each intron).

We analyzed the conservation of intron locations in all *Drd2*, *Drd3*, *Drd4*, and *Actb* genes. For this purpose, our numbering of exons followed the exon numbers of the homologous exons in the following human genes – *DRD2* (HGNC:3023), *DRD4* (HGNC: 3025) and *ACTB* (HGNC:132). For *Drd3* genes, we used the exon numbers from mouse *Drd3*, because the human *DRD3* gene was not representative of the mammalian consensus. In fact, the human *DRD3* gene has only one phase 0 intron between exons 5 and 7, evidently because exon 6 was deleted by recombination between the pair of phase 0 introns that originally flanked exon 6 (see Fig. 2).

### mRNA Isoform-Specific ej-RNAs

Ej-RNAs were computational oligonucleotide sequences that were used to detect a specific mRNA splice junction between two exons. All ej-RNAs consisted of 15 nucleotides from the upstream exon, followed by 15 nucleotides from the downstream exon. These lengths were chosen after control experiments, which showed that these lengths were sufficient to reduce the false positive rate to zero in BLAST searches of RNA-Seq projects (each containing ~ 60 million RNA-Seq reads), in which all matching RNA-Seq reads were downloaded and aligned with the gene in question (see the Quality Control sections in “Results”). Conversely, experiments with longer ej-RNAs returned fewer matches from RNA-Seq libraries (not shown), which was undesirable because that would have reduced the sensitivity of mRNA isoform detection.

We designed ej-RNAs based on sequence information in RefSeq, which was supplemented in some cases by individual mRNA sequences from NCBI or ENSEMBL (European Bioinformatics Institute) databases. All of the ej-RNAs sequences that we used were unique to each species, and were also unique to the gene and splice junction being tested (Supplementary Table S6). More specifically, the *Drd2*_*L*_ ej-RNAs included the last 15 nucleotides of exon 5 joined to the first 15 nucleotides of exon 6, while the *Drd2*_*S*_ ej-RNAs included the last 15 nucleotides of exon 5 joined to the first 15 nucleotides of exon 7 (Supplementary Table S6). In most cases, the *Actb* ej-RNAs included the last 15 nucleotides of exon 3 joined to the first 15 nucleotides of exon 4 (Supplementary Table S7).

As of this writing, *Lithobates (Rana) catesbeiana* (bullfrog) did not have an available genome sequence, but it did have an available brain RNA-Seq library (Supplementary Table S1), together with three published sequences of *Drd2* mRNA isoforms (Nakano et al. 2010), and one beta-actin mRNA sequence (Supplementary Table S7). Starting with this information, we were able to deduce the locations of the exon/intron splice junctions, and design ej-RNAs accordingly (Supplementary Tables S2, S6, S7). These ej-RNA sequences were validated by BLAST searches of *L. catesbeiana* RNA-Seq libraries.

Similarly, *Lithobates (Rana) pipiens* (leopard frog) also did not have an available genome sequence, but did have an available brain RNA-Seq library (Supplementary Table S1). In the absence of any available complete mRNA sequences from *L. pipiens*, we used Geneious software to assemble the *Drd2* (D2-1) mRNA sequence from *L. pipiens* RNA-Seq reads (Supplementary Table S2). Our assembly was validated by comparing the conceptually translated products of the *L. catesbeiana* and *L. pipiens Drd2* mRNA sequences, which shared 99.8% amino acid identity. We also used Geneious software to assemble the beta-actin mRNA sequence from *L. pipiens* RNA-Seq reads (Supplementary Table S7). This assembly was validated by comparing the conceptually translated products of the *L. catesbeiana* and *L. pipiens* beta-actin mRNA sequences, which gave 100% amino acid identity at the unambiguous sites [i.e., not counting ambiguous amino acids ("X") in the *L. catesbeiana* sequence, which corresponded to ambiguous DNA base calls]. We designed ej-RNAs for *L. pipiens Drd2* and beta-actin from the assembled mRNA sequences.

As of this writing, *Notophthalmus viridescens* (eastern newt) did not have an available genome sequence, but it did have an available brain RNA-Seq library (Supplementary Table S1), as well as one mRNA sequence of the *Drd2*_*L*_ isoform (Supplementary Table S2) and one mRNA sequence of beta-actin (Supplementary Table S7). Based on our observations that intron locations were always (*Drd2*) or usually (*Actb*) conserved across species comparisons from lamprey (*P. marinus*) to human (*H. sapiens*), we assumed that these intron locations were also conserved in *N. viridescens*. This assumption was used to design ej-RNAs for *N. viridescens Drd2* and *Actb* (Supplementary Tables S6, S7). These ej-RNAs were validated by subsequent BLAST searches of *N. viridescens* RNA-Seq libraries.

### Quantification of *Drd2*_*L*_ and *Drd2*_*S*_ mRNA Isoforms

We quantified *Drd2*_*L*_* and Drd2*_*S*_ mRNA splicing isoforms and *Actb* mRNA by searching "short read" (RNA-Seq) brain libraries from selected species, using SRA BLAST (Leinonen et al. 2011). We counted all RNA sequence reads that had perfect matches (i.e., 30/30 nucleotide identities, and no gaps) to our ej-RNAs. These criteria were different from, and arguably more stringent than, the criteria used by Barbosa-Morais et al. who utilized junctional sequences with a match of at least 8 bases to each exon, and a maximum total of two mismatches, subject to the additional constraints that the junctional sequence match a single genomic region, and a single gene within the "canonical transcriptome" (Barbosa-Morais et al. 2012). Both of these methods were designed to retrieve transcripts from a single gene, although our quality control approach had the additional feature of being able also to detect the presence of chimeric RNA-Seq reads (see “Results”).

The RNA-Seq read libraries that we searched were digitally archived in the sequence read archives (SRA) of NCBI. For each SRA library tested, we recorded the number of RNA-Seq reads that had perfect matches to our ej-RNAs for that species. We optimized the SRA BLAST search parameters as follows. All searches were configured to use the shortest available word size (*k* = 7 bases), because we found that decreasing the word size increased the number of valid matches that were retrieved, without causing any artifactual (false positive) matches to other genes (see "Quality Control" sections in “Results”). Conversely, increasing the word size to k > 7 did cause a few false negatives, which we attributed to computational issues that may arise when RNA sequences, although otherwise perfectly matched, end before reaching a word boundary (either in the query or in the target sequence). Other BLAST parameters were automatically set to values appropriate for “short input sequences,” due to the lengths of our query sequences and the read lengths in the sequence libraries. These parameter values were as follows: Match score = + 1, Mismatch score = − 3, Gap Initiation Penalty = − 5, Gap Extension Penalty = − 2.

Alternative splicing of the *Drd2* gene was quantified by counting the number of perfect matches in each library to ej-RNAs that were diagnostic for the *Drd2*_*L*_ and *Drd2*_*S*_ isoforms, respectively. As a control, we used a similar process to quantify the numbers of perfect matches to ej-RNAs that were diagnostic for the standard isoform of cytoplasmic β-actin (*Actb*). This enabled us to normalize the expression of each splicing isoform to *Actb*, as we have previously done for quantitative reverse transcriptase polymerase chain reaction (qRT-PCR) experiments (Polesskaya et al. 2007). The counts of properly spliced actin isoforms also provided a useful indication of the quality of each SRA library, because the number of *Actb* reads that we retrieved reflects not only the total number of sequencing reads, but also the average length per read, the frequency of sequence artifacts, and so on.

### Conserved Protein Domains

Protein domains were analyzed for local amino acid sequence conservation, which is a useful indicator of protein structure and function (Fryxell and Meyerowitz 1991). Multiple protein alignments were quantitatively analyzed for local sequence similarity essentially as previously described (Fryxell and Meyerowitz 1991). Briefly, each location in each pairwise comparison (within the multiple protein alignment) was assigned a score of two points for an amino acid identity, one point for a conservative substitution, and zero points for nonconservative substitutions, single gaps, and double gaps (aligned gaps in both proteins being compared). The score for any particular pairwise identity was increased to three points if, and only if, that location was identical in all sequences within the multiple protein alignment. The "net score" at a single location of the multiple alignment was then computed as the average of all possible pairwise comparisons at that location. The "local similarity" of a sliding computational window was computed as the average of the net scores at all locations within the window. The resulting "local similarity" scores were graphed with respect to the sequence location of the center of the window (see Fig. 4). In the experiments reported here, local similarity scores were calculated within a computational window that was 30 amino acids in length.

### Protein BLAST Searches

For Table 1, BLASTP searches of the non-redundant version of Genbank were conducted with parameters adjusted for short query sequences (word size = 2, scoring matrix = PAM30, gap initiation penalty = − 9, gap extension penalty = − 1, significance threshold = E-value of 0.05).

When we used the *Drd2* exon 6 amino acid sequence of the dog (*Canis lupus familiaris*; 29 amino acids) as the query sequence, we obtained significant matches to 1,762 sequences. Of these significant matches, 8 were annotated as synthetic constructs and were discarded because they were not natural sequences. Of the remaining significant BLAST matches, 142 had ambiguous or potentially non-D2 annotations such as "hypothetical protein", "unnamed protein product”, “seven transmembrane helix receptor", "dopamine receptor", "D dopamine receptor", "D3-like" or "bromodomain-containing protein 8". These 142 ambiguously annotated sequences were used as full-length queries for additional rounds of BLASTP. For full-length BLASTP, we used the following parameters: word size = 6, scoring matrix = BLOSUM62, gap initiation penalty = − 11, gap extension penalty = − 1, and significance threshold = E-value of 0.05. For 141 of these query sequences, we found that the vast majority of their full-length matches in each case were annotated as D2 dopamine receptors (plus sometimes a few matches annotated as "hypothetical proteins" etc. as in the first search). We concluded that these 141 query sequences did represent valid D2 dopamine receptors, whose only problem was that their annotation was incomplete. Finally, 1 query sequence was determined to be a chimeric fusion (sequencing artifact) composed of sequences from a D2 dopamine receptor, plus an unrelated protease gene (not shown). This was discarded because it was not a natural sequence.

We also noted that 575 of the above D2 dopamine receptor sequences were isoforms of the same gene(s), or partial sequences from the same gene that had been reported by different authors. These duplicate sequences were not included in the gene counts shown in Table 1.

### Software and Computational Tools

SRA BLAST (Leinonen et al. 2011) was used to search RNA-Seq libraries for perfect matches to ej-RNAs. The locations of exon/intron splice junctions were assessed with the NCBI Spline Tool (Kapustin et al. 2008), as well as the RefSeq gene viewer, with data provided by RefSeq genomes, protein and gene inventories (McGarvey et al. 2015; O’Leary et al. 2016). RNA-Seq assemblies were generated with Geneious Prime 2023.0.4 (Biomatters Inc., Auckland, New Zealand; www.geneious.com). Transmembrane segment prediction by Hidden Markov Models (TMHMM v. 2.0, DTU Health Tech, Denmark Technical University, Lyngby, Denmark) was used to identify protein transmembrane segments. Multiple protein sequence alignments and bootstrapped gene trees were produced with MEGA X (Kumar et al. 2018; Stecher et al. 2020). Sequence conservation in multiple protein alignments was quantified with Local_Conservation_Scan_4 (Fryxell and Meyerowitz 1991). Other statistical tests and graphs were produced with PRISM (GraphPad Software, San Diego, CA).

## Supplementary Information

Below is the link to the electronic supplementary material.

Supplementary file1 (PDF 873 KB)

Supplementary file2 (XLSX 45 KB)

## Data Availability

The isoform-specific read counts, accession numbers of sequences used, ej-RNA sequences used, and other data are available in the Supplementary Tables (see above). Our RNA sequence assemblies of the *Lithobates pipiens Drd2* (D2-1) and beta-actin mRNA sequences will be submitted to Genbank.

## References

[CR1] Adrover MF, Shin JH, Alvarez VA (2014) Glutamate and dopamine transmission from midbrain dopamine neurons share similar release properties but are differentially affected by cocaine. J Neurosci 34:3183. 10.1523/jneurosci.4958-13.2014 24573277 10.1523/JNEUROSCI.4958-13.2014 PMC3935083

[CR2] Barbosa-Morais NL, Irimia M, Pan Q, Xiong HY, Gueroussov S, Lee LJ, Slobodeniuc V, Kutter C, Watt S, Colak R, Kim T, Misquitta-Ali CM, Wilson MD, Kim PM, Odom DT, Frey BJ, Blencowe BJ (2012) The evolutionary landscape of alternative splicing in vertebrate species. Science 338:1587. 10.1126/science.1230612 23258890 10.1126/science.1230612

[CR3] Bertolino A, Fazio L, Caforio G, Blasi G, Rampino A, Romano R, Di Giorgio A, Taurisano P, Papp A, Pinsonneault J, Wang D, Nardini M, Popolizio T, Sadee W (2009) Functional variants of the dopamine receptor D2 gene modulate prefronto-striatal phenotypes in schizophrenia. Brain 132:417. 10.1093/brain/awn248 18829695 10.1093/brain/awn248 PMC2640212

[CR4] Blasi G, Selvaggi P, Fazio L, Antonucci LA, Taurisano P, Masellis R, Romano R, Mancini M, Zhang F, Caforio G, Popolizio T, Apud J, Weinberger DR, Bertolino A (2015) Variation in dopamine D2 and serotonin 5-HT2A receptor genes is associated with working memory processing and response to treatment with antipsychotics. Neuropsychopharmacology 40:1600. 10.1038/npp.2015.5 25563748 10.1038/npp.2015.5 PMC4915265

[CR5] Braasch I (2020) Genome evolution: domestication of the allopolyploid goldfish. Current Biol 30:R812. 10.1016/j.cub.2020.05.073 10.1016/j.cub.2020.05.073 32693075

[CR6] Caporale LH (1999) Chance favors the prepared genome. Ann NY Acad Sci 870:1. 10.1111/j.1749-6632.1999.tb08860.x 10415469 10.1111/j.1749-6632.1999.tb08860.x

[CR7] Centonze D, Gubellini P, Usiello A, Rossi S, Tscherter A, Bracci E, Erbs E, Tognazzi N, Bernardi G, Pisani A, Calabresi P, Borrelli E (2004) Differential contribution of dopamine D2S and D2L receptors in the modulation of glutamate and GABA transmission in the striatum. Neuroscience 129:157. 10.1016/j.neuroscience.2004.07.043 15489038 10.1016/j.neuroscience.2004.07.043

[CR8] Chen Z, Omori Y, Koren S, Shirokiya T, Kuroda T, Miyamoto A, Wada H, Fujiyama A, Toyoda A, Zhang S, Wolfsberg TG, Kawakami K, Phillippy AM, Comparative Sequencing Program NISC, Mullikin JC, Burgess SM (2019) De novo assembly of the goldfish (*Carassius auratus*) genome and the evolution of genes after whole-genome duplication. Sci Adv 5:eaav0547. 10.1126/sciadv.aav0547 31249862 10.1126/sciadv.aav0547 PMC6594761

[CR9] Clarke T-K, Weiss ARD, Ferarro TN, Kampman KM, Dackis CA, Pettinati HM, O’Brien CP, Oslin DW, Lohoff FW, Berrettini WH (2014) The dopamine receptor D2 (DRD2) SNP rs1076560 is associated with opioid addiction. Ann Hum Genet 78:33. 10.1111/ahg.12046 24359476 10.1111/ahg.12046 PMC4013426

[CR10] Cloutier A, Sackton TB, Grayson P, Clamp M, Baker AJ, Edwards SV, Faircloth B (2019) Whole-genome analyses resolve the phylogeny of flightless birds (Palaeognathae) in the presence of an empirical anomaly zone. Syst Biol 68:937. 10.1093/sysbio/syz019 31135914 10.1093/sysbio/syz019 PMC6857515

[CR11] Cohen OS, Weickert TW, Hess JL, Paish LM, McCoy SY, Rothmond DA, Galletly C, Liu D, Weinberg DD, Huang XF, Xu Q, Shen Y, Zhang D, Yue W, Yan J, Wang L, Lu T, He L, Shi Y, Xu M, Che R, Tang W, Chen CH, Chang WH, Hwu HG, Liu CM, Liu YL, Wen CC, Fann CSJ, Chang CC, Kanazawa T, Middleton FA, Duncan TM, Faraone SV, Weickert CS, Tsuang MT, Glatt SJ (2015) A splicing-regulatory polymorphism in DRD2 disrupts ZRANB2 binding, impairs cognitive functioning and increases risk for schizophrenia in six Han Chinese samples. Mol Psychiatry 21:975. 10.1038/mp.2015.137 26347318 10.1038/mp.2015.137

[CR12] Dal Toso R, Sommer B, Ewert M, Herb A, Pritchett DB, Bach A, Shivers BD, Seeburg PH (1989) The dopamine D2 receptor: two molecular forms generated by alternative splicing. EMBO J 8:4025. 10.1002/j.1460-2075.1989.tb08585.x 2531656 10.1002/j.1460-2075.1989.tb08585.x PMC401577

[CR13] Delsuc F, Brinkmann H, Chourrout D, Philippe H (2006) Tunicates and not cephalochordates are the closest living relatives of vertebrates. Nature 439:965. 10.1038/nature04336 16495997 10.1038/nature04336

[CR14] Dolphin AC, Lee A (2020) Presynaptic calcium channels: specialized control of synaptic neurotransmitter release. Neuroscience 21:213. 10.1038/s41583-020-0278-2 32161339 10.1038/s41583-020-0278-2 PMC7873717

[CR15] Felsenstein J (1985) Confidence limits on phylogenies: an approach using the bootstrap. Evolution 39:783. 10.2307/2408678 28561359 10.1111/j.1558-5646.1985.tb00420.x

[CR16] Feng Y-J, Blackburn DC, Liang D, Hillis DM, Wake DB, Cannatella DC, Zhang P (2017) Phylogenomics reveals rapid, simultaneous diversification of three major clades of Gondwanan frogs at the Cretaceous-Paleogene boundary. Proc Natl Acad Sci USA 114:E5864. 10.1073/pnas.1704632114 28673970 10.1073/pnas.1704632114 PMC5530686

[CR17] Ford CP (2014) The role of D2-autoreceptors in regulating dopamine neuron activity and transmission. Neuroscience 282:13. 10.1016/j.neuroscience.2014.01.025 24463000 10.1016/j.neuroscience.2014.01.025 PMC4108583

[CR18] Frank MJ, Hutchison K (2009) Genetic contributions to avoidance-based decisions: striatal D2 receptor polymorphisms. Neuroscience 164:131. 10.1016/j.neuroscience.2009.04.048 19393722 10.1016/j.neuroscience.2009.04.048 PMC2760598

[CR19] Frank MJ, Moustafa AA, Haughey HM, Curran T, Hutchison KE (2007) Genetic triple dissociation reveals multiple roles for dopamine in reinforcement learning. Proc Natl Acad Sci USA 104:16311. 10.1073/pnas.0706111104 17913879 10.1073/pnas.0706111104 PMC2042203

[CR20] Fredriksson R, Schiöth HB (2006) G Protein-coupled receptors in the human genome. In: Rognan D (ed) Ligand design for G protein-coupled receptors. Wiley-VCH Verlag, Weinheim

[CR21] Fryxell KJ (1994) The evolution of the dopamine receptor gene family. In: Niznik HB (ed) Dopamine receptors and transporters: pharmacology, structure and function. Marcel Decker Inc, New York

[CR22] Fryxell KJ (1996) The coevolution of gene family trees. Trends Genet 12:364. 10.1016/s0168-9525(96)80020-5 8855667 10.1016/s0168-9525(96)80020-5

[CR23] Fryxell KJ, Meyerowitz EM (1991) The evolution of rhodopsins and neurotransmitter receptors. J Mol Evol 33:367. 10.1007/bf02102867 1663559 10.1007/BF02102867

[CR24] Gantz SC, Robinson BG, Buck DC, Bunzow JR, Neve RL, Williams JT, Neve KA (2015) Distinct regulation of dopamine D2S and D2L autoreceptor signaling by calcium. Elife 4:e09358. 10.7554/elife.09358 26308580 10.7554/eLife.09358 PMC4575989

[CR25] Graur D (2016) Molecular and genome evolution. Sinauer Associates, Sunderland

[CR26] Herbert A, Rich A (1999) RNA processing in evolution. Ann NY Acad Sci 870:119. 10.1111/j.1749-6632.1999.tb08872.x 10415478 10.1111/j.1749-6632.1999.tb08872.x

[CR27] Houde PW (1988) Paleognathous birds from the early tertiary of the northern hemisphere. Nuttall Ornithological Club, Cambridge

[CR28] Jones DT, Taylor WR, Thornton JM (1992) The rapid generation of mutation data matrices from protein sequences. CABIOS 8:275. 10.1093/bioinformatics/8.3.275 1633570 10.1093/bioinformatics/8.3.275

[CR29] Kapustin Y, Souvorov A, Tatusova T, Lipman D (2008) Splign: algorithms for computing spliced alignments with identification of paralogs. Biol Direct 3:13. 10.1186/1745-6150-3-20 18495041 10.1186/1745-6150-3-20 PMC2440734

[CR30] Khan ZU, Mrzljak L, Gutierez A, De La Calle A, Goldman-Rakic PS (1998) Prominence of the dopamine D2 short isoform in dopaminergic pathways. Proc Natl Acad Sci USA 95:7731. 10.1073/pnas.95.13.7731 9636219 10.1073/pnas.95.13.7731 PMC22740

[CR31] Kim E, Magen A, Ast G (2007) Different levels of alternative splicing among eukaryotes. Nucl Acids Res 35:125. 10.1093/nar/gkl924 17158149 10.1093/nar/gkl924 PMC1802581

[CR32] Kon T, Omori Y, Fukuta K, Wada H, Watanabe M, Chen Z, Iwasaki M, Mishina T, S-iS M, Yoshihara D, Arakawa J, Kawakami K, Toyoda A, Burgess SM, Noguchi H, Furukawa T (2020) The genetic basis of morphological diversity in domesticated goldfish. Current Biol 30:2260. 10.1016/j.cub.2020.04.034 10.1016/j.cub.2020.04.034 32392470

[CR33] Kubale V, Blagotinsek K, Nohr J, Eidne KA, Vrecl M (2016) The conserved arginine cluster in the insert of the third cytoplasmic loop of the long form of the D2 dopamine receptor D_2L_-R acts as an intracellular retention signal. Int J Mol Sci 17:16. 10.3390/ijms17071152 10.3390/ijms17071152 PMC4964525 27447620

[CR34] Kumar S, Stecher G, Li M, Knyaz C, Tamura K (2018) MEGA X: molecular evolutionary genetics analysis across computing platforms. Mol Biol Evol 35:1547. 10.1093/molbev/msy096 29722887 10.1093/molbev/msy096 PMC5967553

[CR35] Leinonen R, Sugawara H, Shumway M (2011) The sequence read archive. Nucleic Acids Res 39:D19. 10.1093/nar/gkq1019 21062823 10.1093/nar/gkq1019 PMC3013647

[CR36] Li C, Fraser NC, Rieppel O, Wu X-C (2018) A Triassic stem turtle with an edentulous beak. Nature 560:476. 10.1038/s41586-018-0419-1 30135526 10.1038/s41586-018-0419-1

[CR37] Lindgren N, Usiello A, Goiny M, Haycock J, Erbs E, Greengard P, Hokfelt T, Borrelli E, Fisone G (2003) Distinct roles of dopamine D2L and D2S receptor isoforms in the regulation of protein phosphorylation at presynaptic and postsynaptic sites. Proc Natl Acad Sci USA 100:4305. 10.1073/pnas.0730708100 12651945 10.1073/pnas.0730708100 PMC153088

[CR38] Loughlin FE, Mansfield RE, Vaz PM, McGrath AP, Setiyaputra S, Gamsjaeger R, Chen ES, Morris BJ, Guss JM, Mackay JP (2009) The zinc fingers of the SR-like protein ZRANB2 are single-stranded RNA-binding domains that recognize 5’ splice site-like sequences. Proc Natl Acad Sci USA 106:5581. 10.1073/pnas.0802466106 19304800 10.1073/pnas.0802466106 PMC2667063

[CR39] Luderman KD, Chen R, Ferris MJ, Jones SR, Gnegy ME (2015) Protein kinase C beta regulates the D2-Like dopamine autoreceptor. Neuropharmacology 89:335. 10.1016/j.neuropharm.2014.10.012 25446677 10.1016/j.neuropharm.2014.10.012 PMC4293343

[CR40] Lyson Tyler R, Bever Gabe S, Scheyer Torsten M, Hsiang Allison Y, Gauthier Jacques A (2013) Evolutionary origin of the turtle shell. Curr Biol 23:1113. 10.1016/j.cub.2013.05.003 23727095 10.1016/j.cub.2013.05.003

[CR41] Marchionni M, Gilbert W (1986) The triosephosphate isomerase gene from maize: introns antedate the plant-animal divergence. Cell 46:133. 10.1016/0092-8674(86)90867-6 3755078 10.1016/0092-8674(86)90867-6

[CR42] Martens GJM, Groenen PMA, Groneveld D, Van Riel MCHM (1993) Expression of the *Xenopus* D2 dopamine receptor. Eur J Biochem 213:1349. 10.1111/j.1432-1033.1993.tb17887.x 8504826 10.1111/j.1432-1033.1993.tb17887.x

[CR43] McGarvey KM, Goldfarb T, Cox E, Farrell CM, Gupta T, Joardar VS, Kodali VK, Murphy MR, O’Leary NA, Pujar S, Rajput B, Rangwala SH, Riddick LD, Webb D, Wright MW, Murphy TD, Pruitt KD (2015) Mouse genome annotation by the RefSeq project. Mamm Genome 26:379. 10.1007/s00335-015-9585-8 26215545 10.1007/s00335-015-9585-8 PMC4602073

[CR44] McGuire AM, Pearson MD, Neafsey DE, Galagan JE (2008) Cross-kingdom patterns of alternative splicing and splice recognition. Genome Biol 9:R50. 10.1186/gb-2008-9-3-r50 18321378 10.1186/gb-2008-9-3-r50 PMC2397502

[CR45] Moyer RA, Wang D, Papp AC, Smith RM, Duque L, Mash DC, Sadee W (2010) Intronic polymorphisms affecting alternative splicing of human dopamine D2 receptor are associated with cocaine abuse. Neuropsychopharmacology 36:753. 10.1038/npp.2010.208 21150907 10.1038/npp.2010.208 PMC3055737

[CR46] Nakano M, Hasunuma I, Okada R, Yamamoto K, Kikuyama S, Machida T, Kobayashi T (2010) Molecular cloning of bullfrog D2 dopamine receptor cDNA: tissue distribution of three isoforms of D2 dopamine receptor mRNA. Gen Comp Endocrinol 168:143. 10.1016/j.ygcen.2010.04.016 20417207 10.1016/j.ygcen.2010.04.016

[CR47] Neve KA (2009) The dopamine receptors. Humana Press, Portland

[CR48] O’Leary NA, Wright MW, Brister JR, Ciufo S, Haddad D, McVeigh R, Rajput B, Robbertse B, Smith-White B, Ako-Adjei D, Astashyn A, Badretdin A, Bao Y, Blinkova O, Brover V, Chetvernin V, Choi J, Cox E, Ermolaeva O, Farrell CM, Goldfarb T, Gupta T, Haft D, Hatcher E, Hlavina W, Joardar VS, Kodali VK, Li W, Maglott D, Masterson P, McGarvey KM, Murphy MR, O’Neill K, Pujar S, Rangwala SH, Rausch D, Riddick LD, Schoch C, Shkeda A, Storz SS, Sun H, Thibaud-Nissen F, Tolstoy I, Tully RE, Vatsan AR, Wallin C, Webb D, Wu W, Landrum MJ, Kimchi A, Tatusova T, DiCuccio M, Kitts P, Murphy TD, Pruitt KD (2016) Reference sequence (RefSeq) database at NCBI: current status, taxonomic expansion, and functional annotation. Nucleic Acids Res 44:D733. 10.1093/nar/gkv1189 26553804 10.1093/nar/gkv1189 PMC4702849

[CR49] Öztürk Z, O’Kane CJ, Pérez-Moreno JJ (2020) Axonal endoplasmic reticulum dynamics and its roles in neurodegeneration. Front Neurosci. 10.3389/fnins.2020.00048 32116502 10.3389/fnins.2020.00048 PMC7025499

[CR50] Pinheiro PS, Mulle C (2008) Presynaptic glutamate receptors: physiological functions and mechanisms of action. Nat Rev Neurosci 9:423. 10.1038/nrn2379 18464791 10.1038/nrn2379

[CR51] Polesskaya OO, Smith RF, Fryxell KJ (2007) Chronic nicotine doses down-regulate PDE4 isoforms that are targets of antidepressants in adolescent female rats. Biol Psychiatry 61:56. 10.1016/j.biopsych.2006.03.038 16814262 10.1016/j.biopsych.2006.03.038

[CR52] Popesku JT, Navarro-Martín L, Trudeau VL (2011) Evidence for alternative splicing of a dopamine D2 receptor in a teleost. Physiol Biochem Zool 84:135. 10.1086/658290 21460524 10.1086/658290

[CR53] Putnam NH, Butts T, Ferrier DE, Furlong RF, Hellsten U, Kawashima T, Robinson-Rechavi M, Shoguchi E, Terry A, Yu JK, Benito-Gutierrez EL, Dubchak I, Garcia-Fernandez J, Gibson-Brown JJ, Grigoriev IV, Horton AC, de Jong PJ, Jurka J, Kapitonov VV, Kohara Y, Kuroki Y, Lindquist E, Lucas S, Osoegawa K, Pennacchio LA, Salamov AA, Satou Y, Sauka-Spengler T, Schmutz J, Shin IT, Toyoda A, Bronner-Fraser M, Fujiyama A, Holland LZ, Holland PW, Satoh N, Rokhsar DS (2008) The amphioxus genome and the evolution of the chordate karyotype. Nature 453:1064. 10.1038/nature06967 18563158 10.1038/nature06967

[CR54] Radl D, Chiacchiaretta M, Lewis RG, Brami-Cherrier K, Arcuri L, Borrelli E (2018) Differential regulation of striatal motor behavior and related cellular responses by dopamine D2L and D2S isoforms. Proc Natl Acad Sci USA 115:198. 10.1073/pnas.1717194115 29255027 10.1073/pnas.1717194115 PMC5776825

[CR55] Radman M, Matic I, Taddei F (1999) Evolution of evolvability. Ann NY Acad Sci 870:146. 10.1111/j.1749-6632.1999.tb08874.x 10415480 10.1111/j.1749-6632.1999.tb08874.x

[CR56] Robertson B, Huerta-Ocampo I, Ericsson J, Stephenson-Jones M, Perez-Fernandez J, Bolam JP, Diaz-Heijtz R, Grillner S (2012) The dopamine D2 receptor gene in lamprey, its expression in the striatum and cellular effects of D2 receptor activation. PLoS ONE 7:e35642. 10.1371/journal.pone.0035642 22563388 10.1371/journal.pone.0035642 PMC3338520

[CR57] Romer AS (1966) Vertebrate paleontology, 3rd edn. The University of Chicago Press, Chicago

[CR58] Ryan TJ, Grant SG (2009) The origin and evolution of synapses. Nat Rev Neurosci 10:701. 10.1038/nrn2717 19738623 10.1038/nrn2717

[CR59] Saitou N, Nei M (1987) The neighbor-joining method: A new method for reconstructing phylogenetic trees. Mol Biol Evol 4:406. 10.1093/oxfordjournals.molbev.a040454 3447015 10.1093/oxfordjournals.molbev.a040454

[CR60] Sasabe T, Furukawa A, Matsusita S, Higuchi S, Ishiura S (2007) Association analysis of the dopamine receptor D2(DRD2) SNP rs1076560 in alcoholic patients. Neurosci Lett 412:139. 10.1016/j.neulet.2006.10.064 17196743 10.1016/j.neulet.2006.10.064

[CR61] Satou Y, Nakamura R, Yu D, Yoshida R, Hamada M, Fujie M, Hisata K, Takeda H, Satoh N (2019) A nearly complete genome of *Ciona intestinalis* type a (c. Robusta) reveals the contribution of inversion to chromosomal evolution in the genus *Ciona*. Genome Biol Evol 11:3144. 10.1093/gbe/evz228 31621849 10.1093/gbe/evz228 PMC6836712

[CR62] Schoch RR, Sues H-D (2015) A middle Triassic stem-turtle and the evolution of the turtle body plan. Nature 523:584. 10.1038/nature14472 26106865 10.1038/nature14472

[CR63] Sedaghat K, Nantel MF, Ginsberg S, Lalonde V, Tiberi M (2006) Molecular characterization of dopamine D2 receptor isoforms tagged with green fluorescent protein. Mol Biotech 34:1. 10.1385/mb:34:1:1 10.1385/MB:34:1:1 16943566

[CR64] Sen SK, Han K, Wang J, Lee J, Wang H, Callinan PA, Dyer M, Cordaux R, Liang P, Batzer MA (2006) Human genomic deletions mediated by recombination between Alu elements. Am J Hum Genet 79:41. 10.1086/504600 16773564 10.1086/504600 PMC1474114

[CR65] Shioda N (2017) Dopamine D2L receptor-interacting proteins regulate dopaminergic signaling. J Pharmacol Sci 135:51. 10.1016/j.jphs.2017.10.002 10.1016/j.jphs.2017.10.002 29107444

[CR66] Shioda N, Yabuki Y, Wang Y, Uchigashima M, Hikida T, Sasaoka T, Mori H, Watanabe M, Sasahara M, Fukunaga K (2017) Endocytosis following dopamine D2 receptor activation is critical for neuronal activity and dendritic spine formation via Rabex-5/PDGFRb signaling in striatopallidal medium spiny neurons. Mol Psychiatry 22:1205. 10.1038/mp.2016.200 27922607 10.1038/mp.2016.200

[CR67] Smith JW, Fetsko LA, Xu R, Wong Y (2002) Dopamine D2L receptor knockout mice display deficits in positive and negative reinforcing properties of morphine and in avoidance learning. Neuroscience 113:755. 10.1016/s0306-4522(02)00257-9 12182883 10.1016/s0306-4522(02)00257-9

[CR68] Smith JJ, Kuraku S, Holt C, Sauka-Spengler T, Jiang N, Campbell MS, Yandell MD, Manousaki T, Meyer A, Bloom OE, Morgan JR, Buxbaum JD, Sachidanandam R, Sims C, Garruss AS, Cook M, Krumlauf R, Wiedemann LM, Sower SA, Decatur WA, Hall JA, Amemiya CT, Saha NR, Buckley KM, Rast JP, Das S, Hirano M, McCurley N, Guo P, Rohner N, Tabin CJ, Piccinelli P, Elgar G, Ruffier M, Aken BL, Searle SM, Muffato M, Pignatelli M, Herrero J, Jones M, Brown CT, Chung-Davidson YW, Nanlohy KG, Libants SV, Yeh CY, McCauley DW, Langeland JA, Pancer Z, Fritzsch B, de Jong PJ, Zhu B, Fulton LL, Theising B, Flicek P, Bronner ME, Warren WC, Clifton SW, Wilson RK, Li W (2013) Sequencing of the sea lamprey (*Petromyzon marinus*) genome provides insights into vertebrate evolution. Nat Genet 45:415. 10.1038/ng.2568 23435085 10.1038/ng.2568 PMC3709584

[CR69] Stecher G, Tamura K, Kumar S (2020) Molecular evolutionary genetics analysis (MEGA) for macOS. Mol Biol Evol 37:1237. 10.1093/molbev/msz312 31904846 10.1093/molbev/msz312 PMC7086165

[CR70] Tunbridge EM, Narajos M, Harrison CH, Beresford C, Cipriani A, Harrison PJ (2019) Which dopamine polymorphisms are functional? Systematic review and meta-analysis of COMT, DAT, DBH, DDC, DRD1–5, MAOA, MAOB, TH, VMAT1, and VMAT2. Biol Psychiatry 86:608. 10.1016/j.biopsych.2019.05.014 31303260 10.1016/j.biopsych.2019.05.014

[CR71] Ule J, Blencowe BJ (2019) Alternative splicing regulatory networks: functions, mechanisms, and evolution. Mol Cell 76:329. 10.1016/j.molcel.2019.09.017 31626751 10.1016/j.molcel.2019.09.017

[CR72] Usiello A, Baik JH, Rouge-Pont F, Picetti R, Dierich A, LeMeur M, Piazza PV, Borrelli E (2000) Distinct functions of the two isoforms of dopamine D2 receptors. Nature 408:199. 10.1038/35041572 11089973 10.1038/35041572

[CR73] Venkatesh B, Erdmann MV, Brenner S (2001) Molecular synapomorphies resolve evolutionary relationships of extant jawed vertebrates. Proc Natl Acad Sci USA 98:11382. 10.1073/pnas.201415598 11553795 10.1073/pnas.201415598 PMC58738

[CR74] Venkatesh B, Lee AP, Ravi V, Maurya AK, Lian MM, Swann JB, Ohta Y, Flajnik MF, Sutoh Y, Kasahara M, Hoon S, Gangu V, Roy SW, Irimia M, Korzh V, Kondrychyn I, Lim ZW, Tay BH, Tohari S, Kong KW, Ho S, Lorente-Galdos B, Quilez J, Marques-Bonet T, Raney BJ, Ingham PW, Tay A, Hillier LW, Minx P, Boehm T, Wilson RK, Brenner S, Warren WC (2014) Elephant shark genome provides unique insights into gnathostome evolution. Nature 505:174. 10.1038/nature12826 24402279 10.1038/nature12826 PMC3964593

[CR75] Veyrunes F, Waters PD, Miethke P, Rens W, McMillan D, Alsop AE, Grutzner F, Deakin JE, Whittington CM, Schatzkamer K, Kremitzki CL, Graves T, Ferguson-Smith MA, Warren W, Marshall Graves JA (2008) Bird-like sex chromosomes of platypus imply recent origin of mammal sex chromosomes. Genome Res 18:965. 10.1101/gr.7101908 18463302 10.1101/gr.7101908 PMC2413164

[CR76] Wang Z, Burge CB (2008) Splicing regulation: From a parts list of regulatory elements to an integrated splicing code. RNA 14:802. 10.1261/rna.876308 18369186 10.1261/rna.876308 PMC2327353

[CR77] Wang Z, Gerstein M, Snyder M (2009) RNA-Seq: a revolutionary tool for transcriptomics. Nat Rev Genet 10:57. 10.1038/nrg2484 19015660 10.1038/nrg2484 PMC2949280

[CR78] Yamamoto K, Fontaine R, Pasqualini C, Vernier P (2015) Classification of dopamine receptor genes in vertebrates: nine subtypes in Osteichthyes. Brain Behav Evol 86:164. 10.1159/000441550 26613258 10.1159/000441550

[CR79] Zalcman G, Federman N, Romano A (2018) CaMKII isoforms in learning and memory: localization and function. Front Mol Neurosci 11:445. 10.3389/fnmol.2018.00445 30564099 10.3389/fnmol.2018.00445 PMC6288437

[CR80] Zhang H, Sulzer D (2012) Regulation of striatal dopamine release by presynaptic auto- and heteroreceptors. Basal Ganglia 2:5. 10.1016/j.baga.2011.11.004 22712055 10.1016/j.baga.2011.11.004 PMC3375990

[CR81] Zhang Y, Bertolino A, Fazio L, Blasi G, Rampino A, Romano R, Lee M-LT, Xiao T, Papp A, Wang D, Sadée W (2007) Polymorphisms in human dopamine D2 receptor gene affect gene expression, splicing, and neuronal activity during working memory. Proc Natl Acad Sci USA 104:20552. 10.1073/pnas.0707106104 18077373 10.1073/pnas.0707106104 PMC2154469

