# Supplementary material for: Convergent Evolution of Two Dopamine Receptor Genes: Repeated Evolution of Exon 6 Skipping in Drd2, and Repeated Deletion of Exon 6 in Drd3
Source: J Mol Evol. 2025 Jun 9;93(3):441–64. doi: 10.1007/s00239-025-10255-7 (PMC12198306; doi:10.1007/s00239-025-10255-7)
Supplement: Supplementary file 1 — Supplementary file1 (PDF 873 KB) [file 239_2025_10255_MOESM1_ESM.pdf]

Journal of Molecular Evolution

Supplementary Material

Convergent evolution of two dopamine receptor genes: repeated evolution of exon 6 skipping in *Drd2*, and repeated deletion of exon 6 in *Drd3*

Michael T. Peglar[1,2] and Karl J. Fryxell[1]**

1. School of Systems Biology, and the Interdisciplinary Program in Neuroscience, George Mason University, Manassas, VA, USA

2. Math, Science, Business, and Engineering Division, Northern Virginia Community College, 8333 Little River Turnpike, Annandale, VA, USA

*Corresponding authors: E-mail: kfryxell@gmu.edu; mtpeglar@gmail.com.

Supplementary Discussion

Most bony fish possess multiple *Drd2* paralogs, but most tetrapods possess only one *Drd2* paralog

Supplementary Fig. S1 is an expansion of the tree in Fig. 1. This also illustrates two branches of D2 dopamine receptor paralogs, which are labeled as D2α and D2β. The D2α branch in this figure included D2 receptors from fish, sharks, and *Petromyzon marinus* (a cyclostome). The D2β branch included only D2 paralogs in the two major subdivisions of bony fish (sarcopterygii and actinopterygii), with minor exceptions (the D2β branch was missing from the genome of *Cynoglossus semilaevis*).

We note that all of the tetrapod species that we examined had at least one D2 receptor in the D2α group (*X. laevis* had two, see Fig. 7). No tetrapod species had D2 receptors in the D2β group, as if the gene encoding D2β had been deleted in the earliest tetrapods (or in an immediately ancestral fish lineage). *Latimeria chalumnae* (coelacanth, a sarcopterygian fish) may be informative in this regard because it is more closely related to tetrapods than it is to actinopterygian fish (Nikaido et al. 2013). The genome of *L. chalumnae* has one D2α receptor and one D2β receptor (Fig. 7). We found that the gene order in the vicinity of the gene encoding *L. chalumnae* D2β was conserved between *L. chalumnae* and *Lepisosteus oculatus* (spotted gar), *Xenopus tropicalis* (western clawed frog), *Alligator mississippiensis* (American alligator), and *Homo sapiens* – but the D2β dopamine receptor was missing from the latter three tetrapod genomes. This suggests that the gene encoding D2β may have been rather specifically deleted in the common ancestor of surviving tetrapod genomes.

Supplementary Methods

Tests of the impact of sequencing errors on the detection of *Drd2* isoforms

In theory, sequencing errors in the genomic sequences and/or RNA-Seq libraries could also have impacted our ability to detect *Drd2* isoforms. Our dataset originated from a diverse array of species and laboratories, and these may have differed in their sequencing error rates. Our isoform detection criteria was relatively stringent (a 30/30 match to ej-RNAs), and that may have increased the likelihood of false negatives (failures to detect relevant RNA-Seq reads). Our dataset contained 29 species, but each fish genome had 2-5 *Drd2* paralogs. In particular, the goldfish (*Carassius auratus*) has 5 *Drd2* paralogs that are annotated in RefSeq. In the course of these experiments, we obtained a cluster of 126 RNA-Seq reads that clearly does represent an additional *C. auratus Drd2* paralog, beyond those that are present in current genome assemblies. This should be interpreted with caution, given that *C. auratus* is allopolyploid and has considerable genetic heterogeneity between strains (Braasch 2020; Kon et al. 2020). Because the strain distribution of this paralog is unclear, and because this paralog was not included in RefSeq, we did not analyze it further.

We also note that the $Drd2_L$ isoform of *Ornithorhynchus anatinus* (platypus) included two distinct 5' splice sites in exon 5, which required separate ej-RNAs for each splice site. All told, we tested 42 different ej-RNAs for $Drd2_L$ (Supplementary Table S6). Likewise, the ej-RNAs for potential $Drd2_S$ isoforms included all of the above genes, plus some species used two 3' splice sites in exon 7, requiring a total of 52 ej-RNAs for $Drd2_S$ isoforms (Supplementary Table S6).

Within this data set, sequencing errors and sequence diversity could hypothetically have caused false negatives in three different ways: (i) sequencing errors in RNA-Seq reads; (ii) errors in the genomic sequence, causing the ej-RNA sequence to fail to match the relevant RNA-Seq reads; or (iii) allelic diversity of the *Drd2* sequence, causing the ej-RNA to fail to match a substantial fraction of the RNA-Seq reads. We will consider each of these possible factors in turn.

The first type of potential false negative would be sequencing errors in the RNA-Seq reads. This would be expected to most often produce a single base mismatch with respect to the ej-RNA sequence (i.e., 29/30 identities). To quantify how often this occurred, we repeated all of our SRA-BLAST searches, and quantified the number of RNA-Seq reads that did have a single base mismatch within the ej-RNA sequence. We found 47 such RNA-Seq reads, all of which were from *Drd2* transcripts of the

corresponding species. Some of these were presumably due to RNA polymerase (transcriptional) errors. But even if we assume that these were all due to RNA sequencing errors, then the comparison to the 2,198 RNA-Seq reads that we did find with perfect matches to the corresponding ej-RNA (i.e., 30/30 identities) yields an estimated average RNA sequencing error rate of 7×10^{-4}, which would have caused about 2% of the relevant *Drd2* exon junctions to fail our requirement for a 30/30 match. Such a small decrease in sensitivity would not have affected any of our conclusions.

The second type of potential false negative would be errors in the genomic sequence. Such errors might have prevented the detection of the corresponding isoform. In this case, the symptom would have been the absence of matching RNA-Seq reads, which was noted for 2 of the 42 $Drd2_L$ ej-RNAs in our initial BLAST experiments. Further investigation revealed that neither were attributable to errors in the genomic sequence. In the first case ($Drd2_L$ gene of *Danio rerio*), all of the relevant RNA-Seq reads differed from the ej-RNA by the same two base substitutions within a single codon, resulting in a single conservative amino acid substitution [ACG (threonine) -> TCA (serine)]. In such a small fish species, the genomic sequence and the RNA-Seq libraries were likely generated from different fish, and those fish may have carried different alleles at this locus. Therefore, we corrected this problem by simply editing the ej-RNA sequence to match the RNA-Seq reads. In the second case, (*drd2l* gene of *Astyanax mexicanus*), we conducted follow-up BLAST experiments to map all RNA-Seq reads that with sequence similarity to this gene (using the complete *drd2l* coding sequence as a BLAST query sequence). The results showed very sparse RNA-Seq reads, none of which covered the relevant exon junctions. In other words, the failure of this particular ej-RNA to match RNA-Seq reads was caused by low (inadequate) levels of gene expression of *Astyanax mexicanus drd2l.* Therefore, this gene was not examined further.

Continuing with the second type of potential false negatives (genomic sequencing errors), these might also occur within exon junctions that were specific to $Drd2_S$ isoforms. Accordingly, it was important to examine the cases in which $Drd2_S$ isoform were not initially detected (17 species and 28 genes; see Fig. 6). All of these genes and species had detectable $Drd2_L$ isoforms, which did match an ej-RNA that crossed the exon 5 - exon 6 boundary. In that sense, the 15 bases of the exon 5 sequence near the exon boundary had already been validated. In order to validate the complete $Drd2_S$ ej-RNAs (exon 5 - exon 7 junction), we also needed to check the 15 bases of the exon 7 sequence. We tested the exon 7 sequence by constructing

exon 6 - exon 7 junction *Drd2*$_L$ ej-RNAs, which should return perfect (30/30) matches to RNA-Seq reads, because these genes and species expressed the *Drd2*$_L$ isoform. This expectation was confirmed, with minor caveats. First, RNA-Seq reads from the salmon (*Salmo salar*) D2-1 gene (see Supplementary Table S6) differed from our exon 6-7 ej-RNA by a single base substitution, but that discrepancy was located in exon 6, and therefore did not indicate a sequencing error in exon 7, which was the issue being tested. Second, RNA-Seq reads showed that *Astyanax mexicanus drd2b* exon 7 actually used two different 3' splice sites that were only 3 bases apart (for *Drd2*$_L$), although only one of these splice sites was annotated. So we designed corresponding *Drd2*$_S$ ej-RNAs to detect both of these splice sites to see whether either were used in *Drd2*$_S$ (see Supplementary Table S6). Third, RNA-Seq reads from elephant shark (*Callorhinchus milii*) *drd2* had an additional 4 codons (12 bases), due to an incorrect annotation in Ref-Seq of the location of the 3' splice site in exon 7 (this annotation was subsequently corrected). This issue was accommodated in our experiments by editing the *C. milii* ej-RNAs accordingly (for both the exon 5-7 junction and the exon 6-7 junction). We found similar ambiguities (i.e., *Drd2*$_L$ splice isoforms that had two 3' splice sites for exon 7 that were 12 bases apart) for the following five genes: *Chrysemys picta DRD2*, *Notophthalmus viridescens Drd2*, *Phascolarctos cinereus DRD2*, *Xenopus laevis drd2.L*, and *Xenopus tropicalis drd2*. We designed additional ej-RNAs, and determined that none of these splice junctions were used in *Drd2*$_S$ (see Supplementary Table S6).

Finally, the third type of potential false negative would have been allelic variation in the sequences corresponding to our ej-RNAs. The symptom in that case would be a one or two base mismatch (29/30 or 28/30 match to the ej-RNA) that was recovered repeatedly from the RNA-Seq libraries. We found only one example of that, in *Danio rerio*, which was corrected by editing the ej-RNAs as described above.

Identification of outlier genes

Four genes were identified as outliers (notwithstanding annotations ranging from "D2-like" to "D4-like", to "*DRD4*") and excluded from our analysis entirely, because they did not have seven transmembrane segments and/or did not group with other dopamine receptors by bootstrap analysis (see supplementary Fig.

S2 and Table S5). None of these genes we identified as outliers contained a conceptually translated sequence similar to *Drd2* exon 6, nor did any of these outliers contain a pair of closely-spaced phase 0 introns that could plausibly be related to exon 6.

Supplementary References

Braasch I (2020) Genome evolution: domestication of the allopolyploid goldfish. Current Biol. 30:R812. https://doi.org/10.1016/j.cub.2020.05.073

Kon T, Omori Y, Fukuta K, Wada H, Watanabe M, Chen Z, Iwasaki M, Mishina T, Matsuzaki S-iS, Yoshihara D, Arakawa J, Kawakami K, Toyoda A, Burgess SM, Noguchi H, Furukawa T (2020) The genetic basis of morphological diversity in domesticated goldfish. Current Biol. 30:2260. https://doi.org/10.1016/j.cub.2020.04.034

Nikaido M, Noguchi H, Nishihara H, Toyoda A, Suzuki Y, Kajitani R, Suzuki H, Okuno M, Aibara M, Ngatunga BP, Mzighani SI, Kalombo HW, Masengi KW, Tuda J, Nogami S, Maeda R, Iwata M, Abe Y, Fujimura K, Okabe M, Amano T, Maeno A, Shiroishi T, Itoh T, Sugano S, Kohara Y, Fujiyama A, Okada N (2013) Coelacanth genomes reveal signatures for evolutionary transition from water to land. Genome Res. 23:1740. https://doi.org/10.1101/gr.158105.113

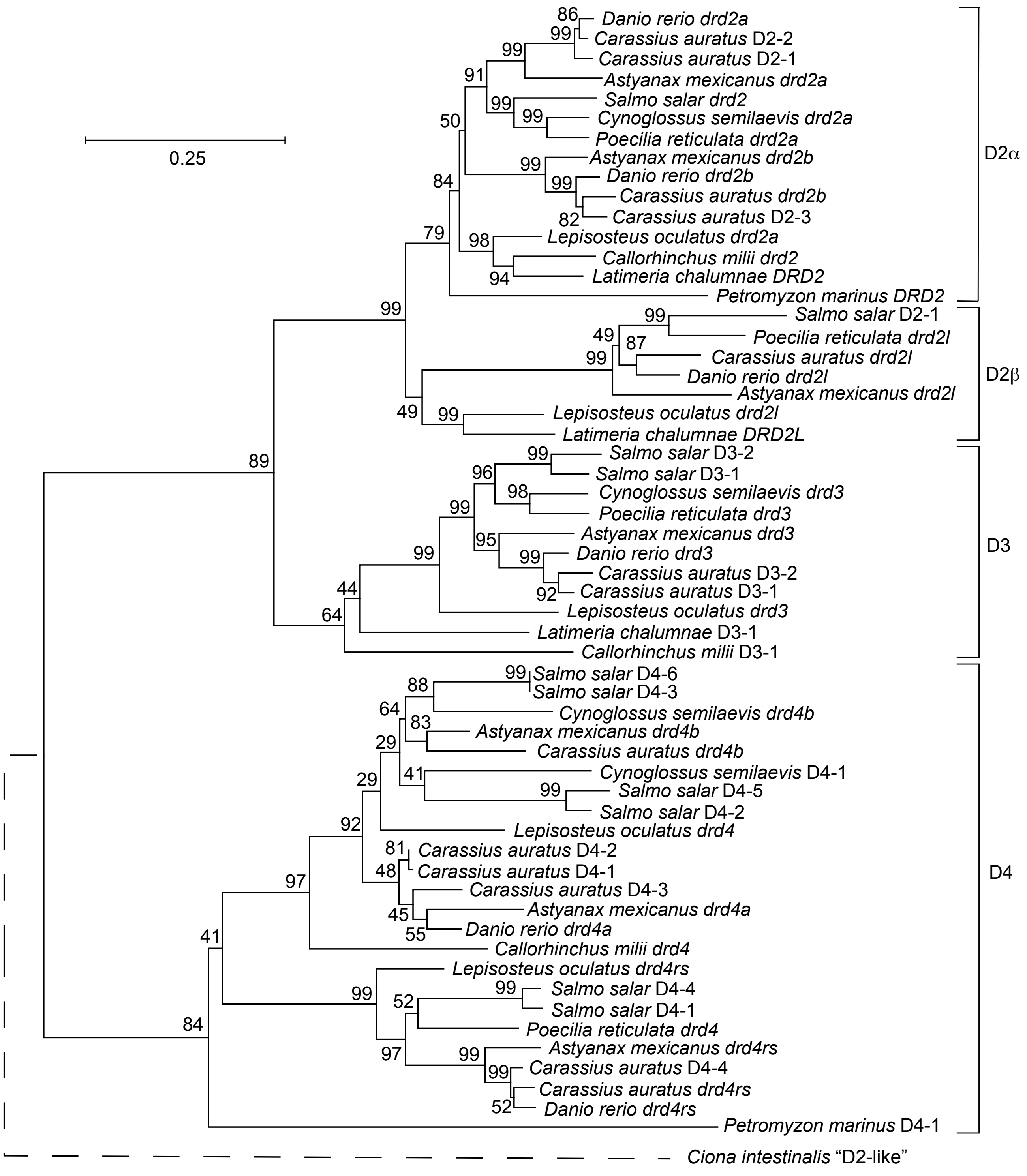

Fig. S1. Protein family tree for D2, D3, and D4 dopamine receptors from tunicate, lamprey, shark and fish genomes. Trees were generated by neighbor-joining. Branch lengths are drawn to scale, in the units of the decimal fraction of amino acid substitutions per site (see scale bar). The resulting trees were bootstrapped to 10,000 iterations. Bootstrap support (in percent) is shown next to each node. The dashed branch indicates the root of the tree, which was not drawn to scale. Gene symbols are shown in italics next to the Latin binomens. If no gene symbol was available at the time of this work, a gene abbreviation was used for the following Ref-Seq accession numbers: *C. auratus*: D2-1, XP_026082110.1; D2-2, XP_026138158.1; D2-3, XP_026109573.1; D3-1, XP_026057526.1; D3-2, XP_026093276.1; D4-1, XP_026057689.1; D4-2, XP_026057915.1; D4-3, XP_026111096.1; D4-4, XP_026088244.1; *C. semilaevis*: D4-1, XP_024912275.1; *C. intestinalis*: D2-like, XP_018667208.2; *P. marinus*: D4-1, XP 032815962.1; *S. salar*: D2-1, XP_014021327.1; D3-1, XP_045558612.1; D3-2, XP_014037004.2; D4-1, XP_014063090.1; D4-2, XP_013980244.1; D4-3, XP_013983146.2; D4-4, XP_045555641.1; D4-5, XP_014025380.2; D4-6, XP_045564906.1; *L. chalumnae*; D3-1, XP_014350627.1. For species common names please see the following Supplementary Tables: Supplementary Table S2 (for D2), Supplementary Table S3 (for D3) and Supplementary Table S4 (for D4).

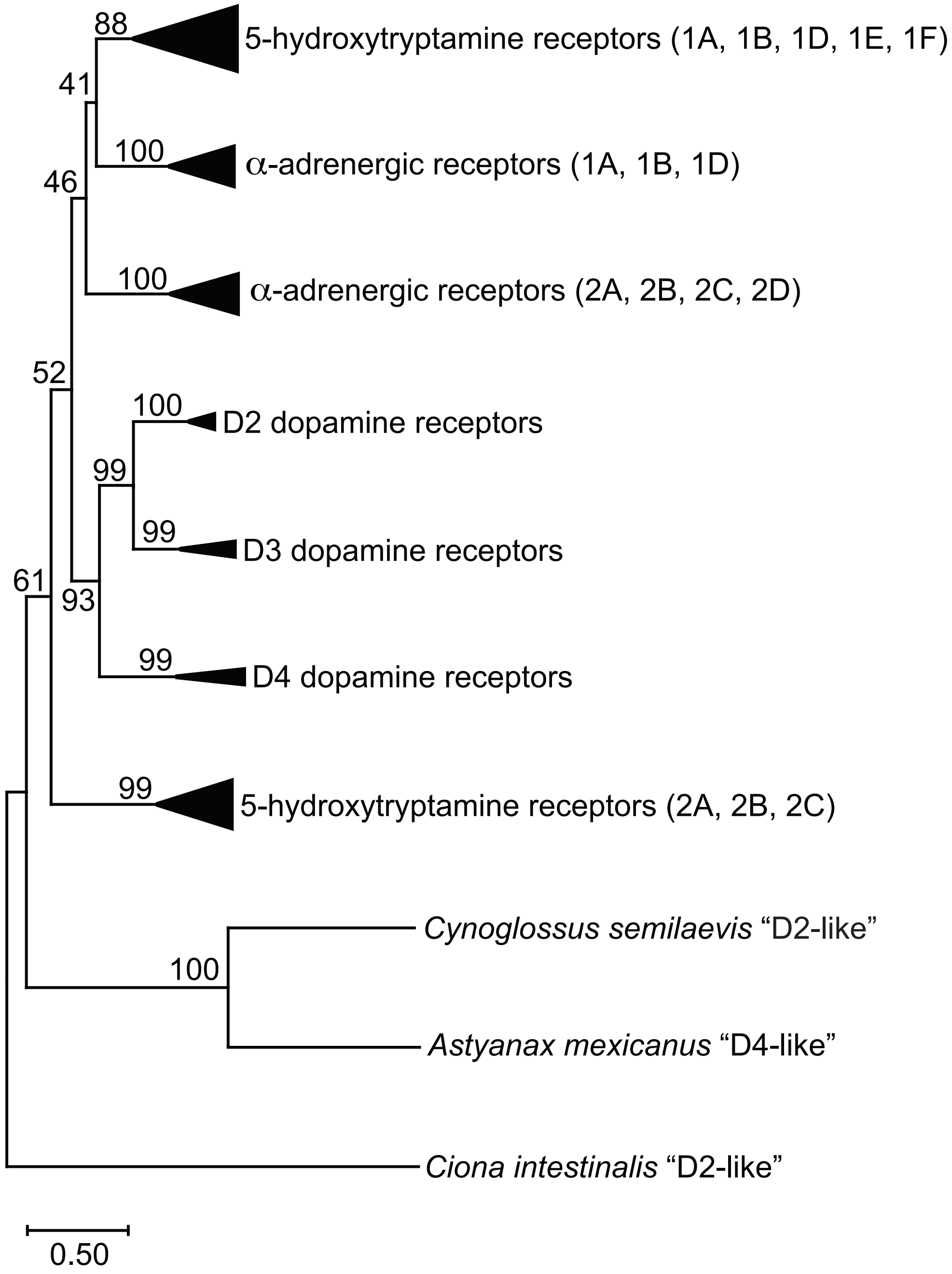

Fig. S2. Protein family tree for putative dopamine receptor outliers, compared to dopamine receptors, 5-hydroxytryptamine receptors, and α-adrenergic receptors from the mouse (*Mus musculus*) and the chicken (*Gallus gallus*). A total of 33 protein sequences were used to generate this unrooted tree by neighbor-joining. Collapsed branches represent chicken and mouse receptors in the indicated groups. Branch lengths were drawn to scale, in the units of the decimal fraction of amino acid substitutions per site (see scale bar). The resulting tree was bootstrapped to 10,000 iterations. The bootstrap support for each branch is shown in percent. Accession numbers for the outliers were as follows: *A. mexicanus* (XP_049342032.1), *C. cynoglossus* (XP_008310418.1), *C. intestinalis* (XP_018667208.2).
